# Structures of mouse and human GITR–GITRL complexes reveal unique TNF superfamily interactions

**DOI:** 10.1038/s41467-021-21563-z

**Published:** 2021-03-02

**Authors:** Feng Wang, Bryant Chau, Sean M. West, Christopher R. Kimberlin, Fei Cao, Flavio Schwarz, Barbara Aguilar, Minhua Han, Winse Morishige, Christine Bee, Gavin Dollinger, Arvind Rajpal, Pavel Strop

**Affiliations:** 1 Discovery Biotherapeutics, Bristol Myers Squibb, Redwood City, CA USA; 2 Discovery Chemistry, Bristol Myers Squibb, Redwood City, CA USA; 3 Tumor Microenvironment Thematic Research Center, Bristol Myers Squibb, Redwood City, CA USA; 4 Genentech Research and Early Development, South San Francisco, CA USA

**Keywords:** Membrane proteins, Cell signalling, Immunotherapy, X-ray crystallography

## Abstract

Glucocorticoid-induced tumor necrosis factor receptor-related protein (GITR) and GITR ligand (GITRL) are members of the tumor necrosis superfamily that play a role in immune cell signaling, activation, and survival. GITR is a therapeutic target for directly activating effector CD4 and CD8 T cells, or depleting GITR-expressing regulatory T cells (Tregs), thereby promoting anti-tumor immune responses. GITR activation through its native ligand is important for understanding immune signaling, but GITR structure has not been reported. Here we present structures of human and mouse GITR receptors bound to their cognate ligands. Both species share a receptor–ligand interface and receptor–receptor interface; the unique C-terminal receptor–receptor enables higher order structures on the membrane. Human GITR–GITRL has potential to form a hexameric network of membrane complexes, while murine GITR–GITRL complex forms a linear chain due to dimeric interactions. Mutations at the receptor–receptor interface in human GITR reduce cell signaling with in vitro ligand binding assays and minimize higher order membrane structures when bound by fluorescently labeled ligand in cell imaging experiments.

## Abstract (web summary)

Glucocorticoid-induced tumor necrosis factor receptor-related protein (GITR) and GITR ligand (GITRL) regulate immune cell activities, including anti-tumor immune responses. Structures and visualization of human and mouse GITR–GITRL complexes offer insight into the architecture of higher-order membrane assemblies, and their signaling.

## Introduction

The tumor necrosis factor (TNF) superfamily of ligands and receptors (TNFSF and TNFRSF) play an important role in cell signaling, activation, and survival. TNFRSF members such as 4-1BB, OX40, CD40, and GITR are present on immune cells and have been shown to regulate immune response^1–4^. As their activity on immune cells can be modulated through agonist and antagonist molecules, TNFRSF members are targets for autoimmune^5^ and cancer indications^6^. Glucocorticoid-induced TNFR-related protein (GITR or TNFRSF18) is a member of the TNFRSF expressed on both effector and regulatory T cells. GITR engagement with GITRL has been shown to drive proliferation and cytokine production of both CD4^+^ Teff and Treg cells^7^, as well as drive antitumor activity of CD8^+^ T cells^8^. Therapeutic agonists have been developed, which may activate Teff cells while depleting Tregs^9^, but agonism with antibodies or GITRL may lead to differing effects^10^. While the structures of the immune complexes between ligand and receptor have been shown for 4-1BB^11,12^, OX40^13^, and CD40^14^, the structure of GITR receptor–ligand complex remains unknown, with only the unbound ligand structures determined^15,16^. A deeper mechanistic insight into GITR ligand–receptor interactions is critical to ensure better understanding of GITR agonists being evaluated in the clinic^17–19^.

Typically, the extracellular domain (ECD) of a TNFSF ligand self-assembles into a threefold symmetric homotrimer through noncovalent hydrophobic interactions^20^. The trimeric ligand engages three receptors, resulting in the assembly of the 3:3 ligand/receptor complex, which leads to the clustering of the receptor cytoplasmic tails and downstream signaling mediated by trimeric TNFR-associated factors (TRAFs)^21^. GITR, a type I transmembrane protein, shares general features of TNFRSF members^20^, but with low (<30%) sequence identity. Upon binding to its ligand, GITRL, GITR produces costimulatory signals regulating T-cell proliferation and effector functions. GITRL, a type II transmembrane protein, bears the smallest ECD (~120 amino acids) and has low (<25%) sequence similarity with other TNF ligands.

Previously reported crystal structures of human GITR ligand (hGITRL) revealed oligomerization interface with little sequence conservation to other TNF ligands. However, this interface is similar to other ligands with aromatic/hydrophobic residues packing at the interface and with a characteristic β-sandwich “jelly roll” topology that self-assembles into homotrimers^15,16^, which may mediate the 3:3 ligand/receptor complex formation found in other TNF members. Although human GITRL shares more than 50% sequence similarity with mouse GITRL, mouse GITRL is a homodimer, suggesting a different 2:2 ligand/receptor stoichiometry. Due to a lack of structural information of these receptors and receptor–ligand complexes, it is challenging to understand how these differences might affect their function.

In this study, we successfully express and purify mouse and human GITR proteins, reconstitute GITR–GITRL complexes for biophysical characterization, and determine the crystal structures of human and mouse GITR–GITRL complexes. Similar to 4-1BB complexes, our studies demonstrate the assembly of a 3:3 human GITR–GITRL complex and a 2:2 mouse GITR–GITRL complex. Our structures show that GITR receptor assembles into a unique structure among TNF superfamily members, which is conserved in both human and mouse. The C-terminal receptor–receptor interactions may play an important role mediating GITR–GITRL complex clustering and GITR network formation on the cell surface.

## Results

### Features of the receptor–ligand structure

Wild-type sequence of human GITR is cysteine rich with 19 cysteines in 137 amino acids in the ECD and expresses recombinantly at very low levels (possibly due to an unpaired cysteine in the sequence). A variant C57S mutant which removed an unconserved free cysteine (Fig. S1) showed improved expression. Therefore, we used hGITR C57S for following studies, referred to as hGITR for these structural and biophysical studies. We subsequently expressed and purified the hGITRL, for cocrystallization with its receptor^15,16^. The crystal structure of human GITR–GITRL complex is solved at 2.96 Å. Similarly, we identified a mouse GITR variant C55S that can be produced recombinantly, referred to as mGITR for these structural and biophysical studies. We expressed mouse GITRL and determined the 3.2 Å structure of the dimeric mGITR–mGITRL complex to compare with the human complex (Table 1, Fig. S2).

**Table 1:** Data collection and refinement statistics (molecular replacement).

	hGITR/GITRL	mGITR/GITRL
Data collection		
Space group	I 2 3	P1 21 1
Cell dimensions		
*a*, *b*, *c* (Å)	168.42, 168.42, 168.42	70.54, 64.82, 81.23
α, β, γ (°)	90, 90, 90	90, 91.23, 90
Resolution (Å)^a^	37.66 − 2.956 (3.062 − 2.956)	35.53 − 3.207 (3.321 − 3.207)
*R*_sym_ or *R*_merge_^a^	0.1407 (1.422)	0.070 (1.233)
*I*/*σI*^a^	20.83 (2.46)	8.3 (1.2)
Completeness (%)^a^	99.92 (100)	90.6 (49.0)
Redundancy^a^	20.5 (21.0)	3.3 (3.3)
Refinement		
Resolution (Å)	2.96	3.20
No. reflections	16801	9120
*R*_work_/*R*_free_	0.22/0.25	0.24/0.28
No. atoms	3442	3641
Protein	3386	3655
Ligand/ion	56	70
B factors	99.46	155.45
Protein	98.94	154.58
Ligand/ion	131.35	200.39
R.m.s. deviations		
Bond lengths (Å)	0.003	0.003
Bond angles (°)	0.67	0.71

^a^Values in parentheses are for highest-resolution shell.

**Fig. 1:**
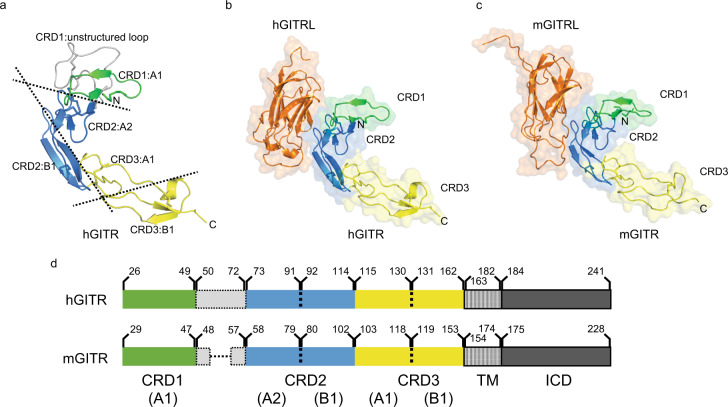
Modular representation of human and mouse GITR. **a** TNFR modular representation of human GITR CRD1 (shown in green), CRD2 (blue), and CRD3 (yellow). CRD1 is unstructured in crystal structure, model of unstructured loop is depicted in white. Individual human GITR–GITRL (**b**) and mouse GITR–GITRL (**c**) heterodimers have similar topology and CRD2-receptor interactions (1:1 receptor–ligand, GITRL in orange). In the domain map (**d**), the resolved regions of the extracellular-domain structure are shown in solid colors, while the unresolved noncanonical loop insertion in CRD1 is boxed (TM transmembrane, ICD intracellular domain). The structured CRD regions are similar between human and mouse. Source data are provided as a Source Data file.

The crystal structure allows us to examine GITR and its interactions, and compare with other TNFRSF members. When viewed alone, hGITR is comprised of three cysteine-rich domains (CRDs) (Fig. 1a), made up of modules as previously described for TNF family members^22^. Prior to resolving this GITR structure, it was noted its CRD1 and CRD2 domains were not homologous to other TNFRSF members^23^, but here we see the arrangement of canonical modules comprising the majority of domains. When comparing the human and mouse receptors, it is apparent that despite interacting with ligands of different stoichiometry, human and mouse GITR are remarkably similar (Fig. 1b, c).

In comparing the receptors, starting at the N-terminus, we note GITR CRD1 is composed of an A1 module, followed by a noncanonical loop between CRD1 and CRD2 (Fig. 1d). The loop insertion in hGITR CRD1 does not have resolved density in our experimental data, and this loop is likely disordered, but is modeled here to illustrate its position. Notably, this loop contains likely disulfide bonds to both the CRD1 A1 subunit and CRD2 A2 subunits based on cysteine locations on the domains. Despite the lack of sequence conservation in GITR CRD1, mouse and human receptors are structurally similar in this region aside from the aforementioned loop.

**Fig. 2:**
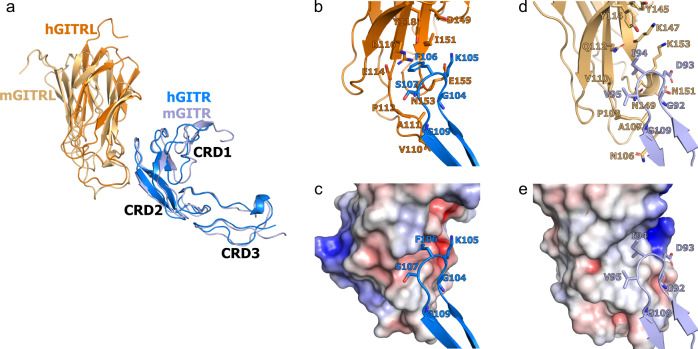
Comparison of human and mouse GITR–GITRL interfaces. **a** Human and mouse GITR interact similarly with their cognate receptors (GITR shown in blue, GITRL in orange). Close view of 1:1 ligand–receptor interfaces are shown for human cartoon (**b**) and electrostatic surface (**c**) and mouse cartoon (**d**) and electrostatic surface (**e**). While having similar overall structure, the interfaces have low sequence conservation, with steric and electrostatic differences.

CRD2 is comprised of A2 and B1 modules, and its B1 module is the only portion of the receptor seen interacting with the ligand in our structure (Fig. 2a). When examining interactions of the hGITR CRD2 binding interface (Fig. 2b), F106 makes the majority of the buried interface, while G104 and G109 allow close contacts between the loop that extends from GITR to contact the membrane-distal portion of GITRL. There is a cavity of negative potential from hGITRL D149 and E155 that positively charged K105 is positioned near. mGITR does not have a buried phenylalanine, but rather an isoleucine, and the glycines are conserved (Fig. 2c). mGITRL has basic charges (K147 and K153), while mGITR has a negative charge (D93), but this electrostatic interaction does not appear as favorable as hGITR. Generally, the small interface (~400 Å^2^) lacks conserved complementary charges or extensive hydrophobic packing (Fig. S3).

**Fig. 3:**
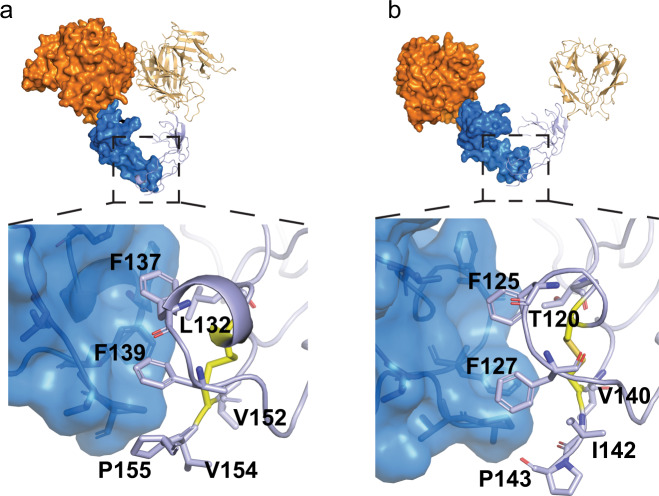
C-terminal receptor–receptor interactions are conserved between human and mouse. Both human (**a**) and mouse (**b**) GITR show receptor–receptor interactions (shown in blue, GITRL in orange). Closer examination shows phenylalanine packing at a hydrophobic homodimeric interface in both human and mouse receptors.

In CRD3, surprisingly, we find mouse and human receptors share a conserved membrane-proximal receptor–receptor interface, forming receptor homodimers between separate ligand–receptor complexes (Fig. 3a, b). This interface occurs in the B1 module of the CRD3. This unexpected GITR receptor–receptor interaction is driven by F137 and F139 in human (Fig. 3a) (F125 and F127 in mouse, Fig. 3b), symmetrically packing against each other at the hydrophobic C-terminal interface. This interface is also small, ~350 Å^2^ for human and ~240 Å^2^ for mouse.

**Fig. 4:**
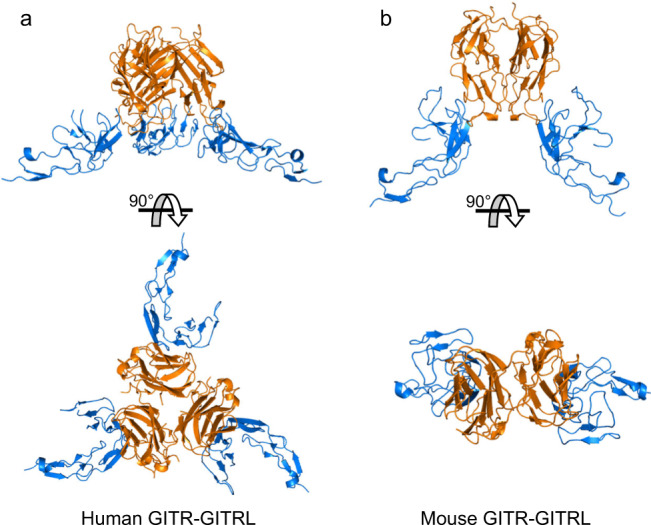
Overview of human and mouse ligand and receptor complexes. Human GITR–GITRL forms a 3:3 receptor–ligand complex (**a**), while mouse GITR–GITRL forms a 2:2 receptor complex (**b**). The ligand is colored orange and the receptor is blue.

Strikingly, both dimeric mouse and trimeric human ligands interact similarly with their receptors, but have 2:2 or 3:3 interactions, respectively (Fig. 4a, b). In comparing the bound ligand structures with previously reported unbound mouse and human ligand structures^16,24^, there is no structural change upon receptor binding for mouse GITRL dimer (Fig. S4a), nor human GITRL trimer (Fig. S4b). Unlike other TNFRSF receptor–ligand structures, each GITR receptor contacts only one ligand subunit and does not make contacts at the ligand–ligand interface. This small single receptor–ligand interface allows for conservation of the receptor–ligand interface, regardless of ligand forming a dimer or trimer.

**Fig. 5:**
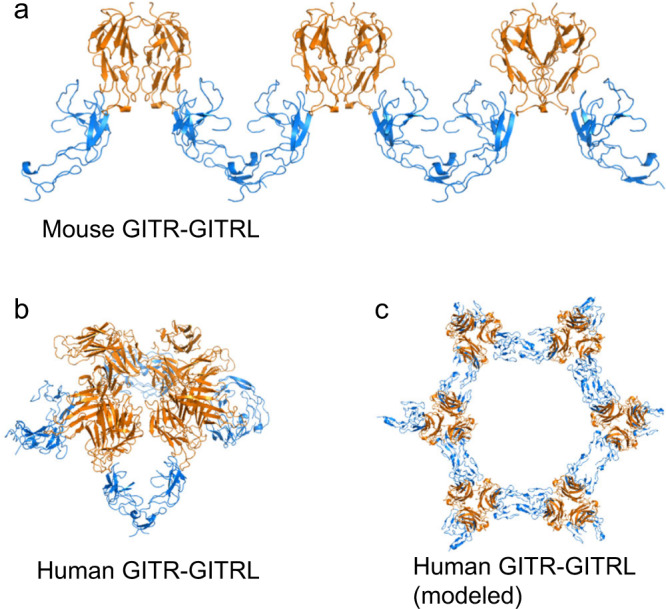
Both human and mouse GITR can form higher-order structures. **a** mGITR can form linear planar networks when interacting with mGITRL. **b** In the crystal structure, hGITRL forms tetrameric trimer complexes (four hGITRL trimers interacting with four hGITR pairs). **c** If modeled as a planar network on the surfaces of opposing cells, human GITR–GITRL creates branching hexamers consisting of six ligand trimers and six receptor trimers. GITRL depicted in orange, GITR in blue.

When considering the stoichiometry of the receptor–ligand complex in the context of the observed receptor–receptor interactions, the overall effect is in the crystallographic assembly mouse receptor–ligand complexes form a linear chained structure (Fig. 5a), while human receptor–ligand complexes form a tetrameric assembly (Fig. 5b). The tetrameric assembly we observe might be an artifact of crystallography, as it has strong curvature and packs as a sphere (Fig. S5). When instead modeled as extended ligand–receptor complexes with ligands in the same plane, as they would be on opposing a cell surfaces, the trimeric human receptor–ligand complexes have potential to form a branched hexameric network (Fig. 5c). This receptor–receptor homodimer is mediated by the dramatic “C” shape of GITR, where only CRD2 interacts with the ligand, while CRD3 curves away at a 90° angle to interact with a neighboring receptor.

In the C-terminus of the ligand, we see the previously reported strand swapping in mouse GITRL, but not in human GITRL, when ligands are complexed with their cognate receptors. It has been observed that a C-terminal deletion in mouse GITRL expressing cells results in increased GITR signaling, possibly because without strand swapping mouse GITRL can form a trimer to signal mouse GITR^25^ similar to the human complex, consistent with our results of a similar ligand–receptor interface between mouse and human GITR–GITRL. To better understand the composition of the receptor and ligands we observed in the crystal structure, we also performed biophysical characterization to determine the stoichiometry in solution.

### Biophysical characterization of human and mouse GITR–GITRL complexes

To assess the stoichiometry of receptors and ligands, we performed size-exclusion chromatography (SEC) coupled to multiangle light scattering (MALS) to experimentally determine molar mass of the species in solution. Analysis of hGITR and mGITR gave a molar mass of 19.7 and 17.9 kDa respectively, indicating monomers (Fig. S6a, b). SEC–MALS analysis of hGITRL showed an experimental molar mass of 45 kDa consistent with a trimeric ligand (Fig. S6c). Analysis of mGITRL indicates a molar mass of 44 kDa (Fig. S6d), larger than predicted mass of 35 kDa for mGITRL dimer, possibly from resistance to endo H cleavage (see “Methods”). Nevertheless, the result agrees with previously reported biophysical characterization and crystal structure of mGITRL dimer. Due to the observed weak interaction, SEC–MALS analysis of the ligand–receptor complexes was unclear.

To directly measure the affinity between ligand and receptor, surface-plasmon resonance (SPR) analysis was used, with the ligand bound to the surface and the receptor flowed over, to prevent avidity prone interactions which may arise when multimeric analyte is flowed over the chip surface. Our data indicate a weak 1:1 receptor–ligand binding affinity for both human and mouse (>5 μM for human and >18 μM for mouse, Fig. S7a, b). This is in agreement with previously published murine^24^ and human^16^ data, but lower than reported affinity for human complexes which reversed the orientation of ligand and receptor and measured the interaction under avidity prone conditions^15^.

To further assess the stoichiometry of the human receptor and ligand, we performed analytical ultracentrifugation (AUC). Sedimentation coefficients of hGITRL and hGITR were measured at 3.7 and 2.14 S, respectively, confirming the observation that hGITRL self-assembles as a homotrimer and hGITR stays as a monomer in solution (Fig. S8a, b). For the complex, hGITR and GITRL were mixed at a molar ratio of 5:3 receptor to ligand, then subjected to SEC. AUC analysis of the complex showed a main peak coefficient of 4.98 S matching the predicted coefficient of a 3:3 receptor: ligand complex (Fig. S8c).

### Validation of the receptor–ligand interface

**Fig. 6:**
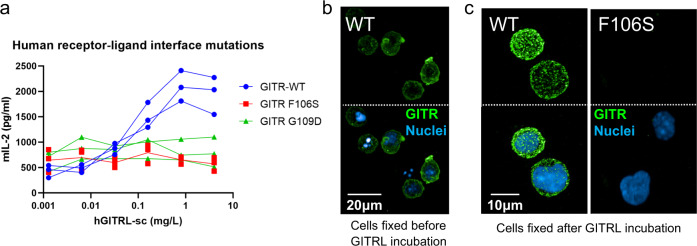
Human receptor mutations which disrupt the receptor–ligand interface. **a** mIL-2 secretion upon hGITRL stimulation with hGITR cell lines expressing WT, F106S, or G109D variants (*n* = 3; independent experiments performed six times). **b** Confocal imaging of WT cells fixed before staining with fluorescently labeled hGITRL-sc trimers shows diffuse surface GITR. **c** WT and F106S mutant hGITR expressing cell lines fixed after staining with fluorescent hGITRL-sc trimers reveals discrete GITR puncta on WT cell surface, but no hGITRL-sc binding of F106S cells. This imaging experiment was performed once, cells plated in duplicate. Source data are provided as a Source Data file.

Our GITR–GITRL complex structures reveal a unique receptor CRD2-ligand binding interface. Two residues from CRD2 were identified as “critical” for ligand binding, F106 and G109. In order to validate our structural analysis, two single mutants (F106S and G109D) were designed to test their impact on the GITR-mediated signaling pathway. We first examined the effect of mutating these residues in a cell-based IL-2 reporter assay. A mouse T-cell line, 3A9, was engineered to express wild-type human GITR and the two human GITR single mutants (F106S or G109D, Fig. S9b). Note, the cell lines for hGITR functional studies do not contain the C57S mutation used to generate recombinant protein for the biophysical studies. The three cell lines were stimulated with native GITRL trimer and the level of secreted IL-2 was measured by ELISA. As expected, the two cell lines with single receptor mutation at the ligand interface failed to respond to native GITRL trimer stimulation when compared to wild-type receptor (Fig. 6a), suggesting these residues on the receptor–ligand interface are essential for binding and downstream signaling.

To provide additional evidence of the effect of receptor mutations on signaling, we performed confocal high-content imaging to visualize GITR clustering on the cell surface. The cell line expressing wild-type hGITR was first fixed, and then treated with fluorescently labeled single chain trimerized hGITRL (hGITRL-sc) for detecting the basal receptor expression, which showed diffuse staining on the cell surface (Fig. 6b). When the same cell line was incubated with labeled hGITRL for 15 min before fixation, it showed a staining pattern of distinct membrane puncta (Fig. 6c, left panel), suggesting receptor clustering upon ligand engagement. This signal was almost completely lost in the cells expressing GITR-F106S mutant (Fig. 6c, right panel). We confirmed by FACS and anti-GITR antibody staining that GITR-F106S expressed at similar level as wild-type GITR (Figs. S9b and S10), suggesting the signal lost in GITR-F106S cells is due to failure of ligand binding. Taking the cell-based assay and high-content imaging results together, we confirmed that the ligand–receptor interface identified from the GITR–GITRL crystal structure are essential for GITR-mediated signal cascade.

### Validation of the receptor–receptor interface and effects on membrane clustering

One unique observation from both human and mouse GITR–GITRL complex structures is the conserved membrane-proximal phenylalanine mediated receptor–receptor interface between separate ligand–receptor complexes. Through modeling, we observe human GITR–GITRL complex may form a hexameric “honeycomb-like” network via receptor dimerization through CRD3, which we hypothesize to be the key mechanism of receptor clustering for amplifying downstream cell signal. To test our hypothesis, two phenylalanine residues in hGITR CRD3, F137 and F139, were mutated to alanine (GITR-AA), aspartic acid (GITR-DD), or arginine (GITR-RR), to abolish the hydrophobic receptor–receptor membrane-proximal interface.

**Fig. 7:**
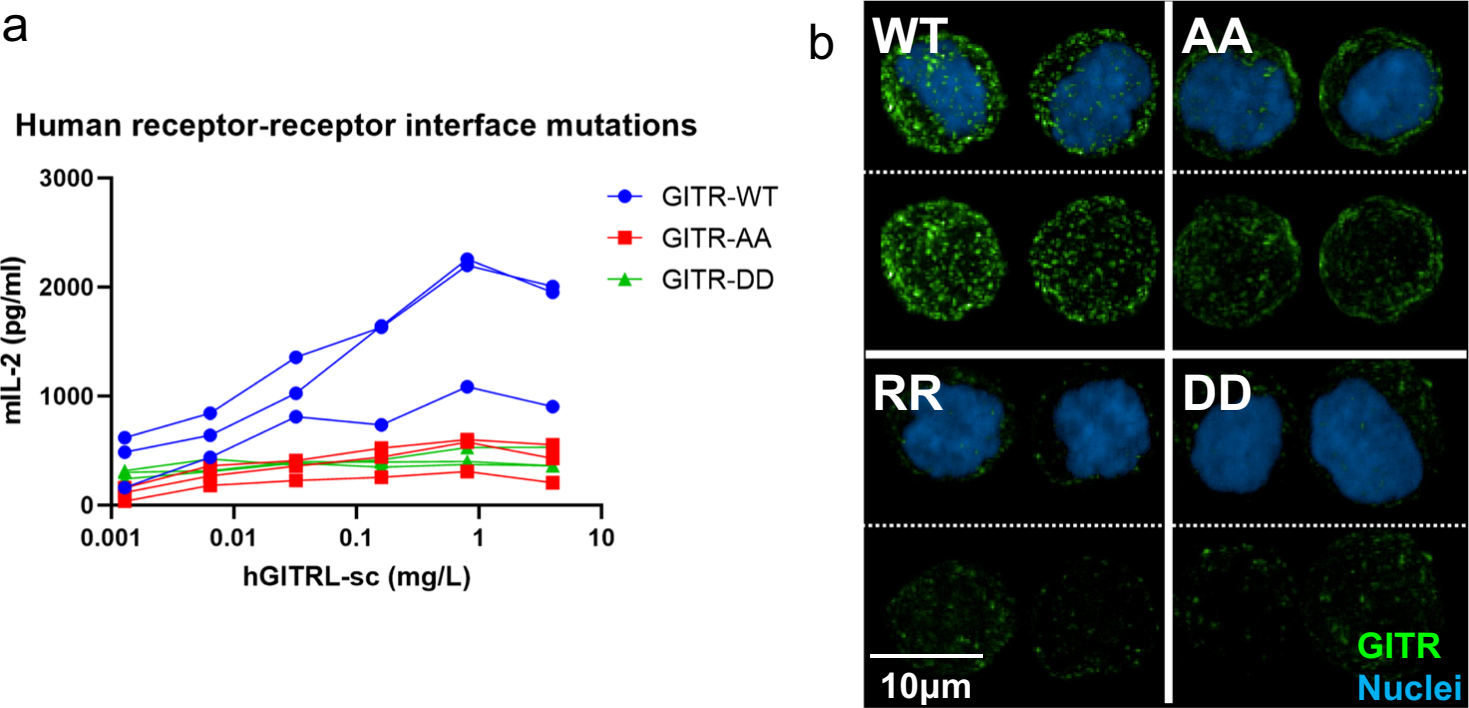
Human receptor mutations which disrupt the receptor–receptor interface. **a** mIL-2 secretion upon hGITRL stimulation with hGITR cell lines expressing WT GITR, AA, and DD variants (*n* = 3; independent experiments performed three times). **b** WT cell line (top left) shows distinct puncta with fluorescently labeled hGITRL-sc trimer staining, while AA cell line (top right) shows reduced receptor binding and clustering. DD and RR (bottom panels) show near complete loss of receptor staining with hGITRL-sc trimers. This imaging experiment was performed once, cells plated in duplicate. Source data are provided as a Source Data file.

Mouse T-cell 3A9 cell lines expressing GITR-AA or GITR-DD were tested in the IL-2 reporter assay. All cell lines had expression comparable to WT, determined by anti-GITR antibody staining (Figs. S9b and S11a). Our results showed interference of receptor–receptor interaction made the cells largely unresponsive to stimulation with the native GITRL trimer: the double alanine mutant reduced the IL-2 secretion level by 78% compared with wild-type GITR (Fig. 7a). Mutants which instead replace phenylalanine with charged aspartic acid residues have almost no IL-2 secretion, indicating abolished GITR signaling due to loss of the CRD3 receptor–receptor interface (GITR-RR similarly does not respond to hGITRL-sc, but has high background mIL-2 level). Based on the structural model, we speculate that loss of this homodimeric interface prevents higher-order structures from forming on the membrane surface, thereby reducing signaling that occurs with crosslinking with either the ligand trimer or agonist antibody.

We then assessed the impact of mutating the receptor–receptor interface on GITR assembly upon GITRL trimer stimulation. 3A9 cells expressing AA, RR, or DD mutants were incubated with GITRL trimer and then fixed for imaging. All cell lines have similar levels of receptor expression (Fig. S11a–c). Intriguingly, the cell line expressing double alanine mutations showed staining ranging from diffuse basal levels to fewer puncta with lower fluorescent signal (Fig. 7b, top right panel). The reduced punctate staining with lower fluorescent signal suggests double alanine replacement might not completely abolish GITR homodimerization and signaling, consistent with weak activity in the IL-2 reporter assay. GITR-RR and GITR-DD showed near complete loss of receptor staining (Fig. 7b, bottom panels; Fig. S11d, e), consistent with loss of function in the IL-2 assay. Because of weak 1:1 GITR–GITRL affinity, reduced receptor–receptor clustering might destabilize interactions on the cell surface where receptor multimers could enhance binding avidity. This is consistent with the reduction or loss in hGITRL-sc fluorescent staining in the AA, RR, and DD mutants.

### Comparison to other TNFRSF members

**Fig. 8:**
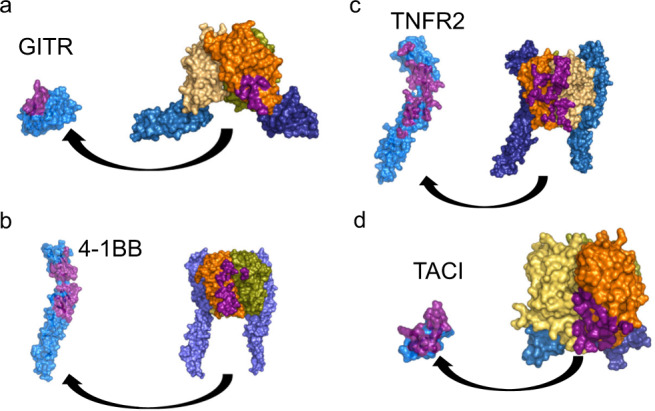
Comparison of TNF–TNFR complexes highlights minimal GITR interface. **a** hGITR–hGITRL (380 Å^2^ interface). **b** h4-1BB–h4-1BBL (6MGP, 950 Å^2^ interface). **c** hTNFa–hTNFR2 (3ALQ, 1330 Å^2^ interface). **d** mAPRIL–hTACI (1XU1, 880 Å^2^ interface). Interactions are shown for surfaces of trimeric ligands (orange, tan, and olive) and receptors (light blue, deep teal, and marine), with single receptor removed to show receptor–ligand interface (purple).

While the hGITR–hGITRL structure demonstrates fundamental characteristics of the TNF and TNFR superfamilies, with its trimeric ligand and 3:3 receptor–ligand stoichiometry, several aspects of the complex set it apart from other family members. For instance, the interaction between GITR and its ligand differs substantially from the canonical TNF receptors that bind at the interface between ligands within a trimer, as seen with the prototypical TNFa-TNFR2 complex^26^. Of the previously solved TNF and TNFR superfamily structures, only the h4-1BB receptor–ligand complex^11^, in which one ligand dominates the binding interface while the second ligand has nonessential contacts, deviates from this trend (Fig. 8). By contrast, GITR binds completely independently of adjacent ligand members. In addition, GITR binding occurs at a distal portion of the ligand trimer, at loops CD and GH, rather than along the trimer.

This is reminiscent of the interaction between the TNF ligand APRIL and its two receptors, TACI and BCMA, though like other TNFRSF members, they bind at the cleft between two adjacent ligands^27^. Furthermore, the average buried interface area between GITR and GITRL is only 378 Å^2^ (calculated with PISA). This is substantially smaller than all other known examples of TNF receptor–ligand complexes, with buried surface area ranging from ~800–1300 Å^2^. The small binding interface likely accounts for the relatively weak (>5 μM) interactions measured between GITR and GITRL. GITR binding occurs at the B1 module of its CRD2 domain, while other TNFRSF members that have some structural similarity utilize the A1/A2 module from their CRD2 domains, as well as contacts from additional domains to bind ligand (Fig. S12).

Consistent with previously solved structures of mGITRL^24,25^, our murine complex structure shows the same dimeric arrangement of the ligand, while preserving the receptor–ligand interface seen in hGITR–hGITRL. The only other known TNFSF member to show this dimeric arrangement is mouse 4-1BBL. Other examples of mouse TNF ligands such as RANKL, OX40, and APRIL all show the canonical trimeric ligand assembly^13,28–30^. Though both m4-1BBL and mGITRL form dimers, the mechanisms driving dimerization are different. In m4-1BBL, a disulfide bond at the base of the dimer covalently locks the ligands in this arrangement^20^, while in mGITRL, dimerization is driven by a strand swap at the C-terminus between ligands^24,31^. Of note, recent work shows that m4-1BBL undergoes a significant shift upon receptor binding, with one ligand protomer rotating relative to the other caused by conformational changes in the loop containing the intermolecular disulfide bond, pulling the ligands closer. In contrast, the loop rearrangements that occur upon GITR binding GITRL do not result in any allosteric affects at the dimer interface and the apo mGITRL dimer and receptor-bound mGITRL dimer are effectively superimposable (Fig. S13).

One of the most striking features shared between both our human and mouse GITR–GITRL complex structures is the apparent dimerization of GITR molecules from adjacent complexes at the interface between CRD3 (Fig. 3a, b). This same interaction is seen in both species, in different crystal forms and in both the trimeric and dimeric arrangements of the complex, suggesting this interface likely plays a critical role in GITR signal transduction, evidenced by our cell assays and imaging studies. This interface may be largely unique to GITR. A PSI-BLAST search across TNFSFR sequences, to identify other family members with hydrophobic C-terminal motifs at this location only finds OPG, a soluble decoy receptor for RANKL, which interestingly also has a bent shape curved away from the ligand. The hydrophobic motif in this OPG CRD domain has been hypothesized to interact intramolecularly with the flanking death domain 1^32^, but has not been tested for CRD–CRD interactions.

Examples of TNF receptor dimerization have been seen in other family member such as TRAIL receptor 1, CD40, Fas, and TNFR1^33^. Of these, TNFR1 is perhaps the best understood example, with biochemical data and crystal structures identifying a preligand assembly domain (PLAD) in the N-terminal CRD1 that mediates ligand-independent receptor clustering (Fig. S14)^33,34^. The parallel arrangement of the TNFR1 homodimer still permits ligand binding and allows for the formation of higher-order receptor clustering and signal amplification. The presence of a PLAD-like interaction in both mouse and human GITR suggests that GITR uses a similar strategy; TNFR1 interacts with nearby receptors using its membrane-distal CRD1 domain, while GITR uses the membrane-proximal CRD3.

## Discussion

In this work, we describe the structure of GITR bound to its native ligand. To isolate these molecules in quantities needed for crystallographic studies, we identified unpaired cysteines that allowed the receptor to be expressed and purified. The receptor is monomeric in solution, while the ligands are trimeric or dimeric in solution for human and mouse respectively. Weak affinity of receptor for ligand was shown through SPR.

GITR has been a therapeutic target and these structures might aid in translating preclinical murine models to human therapeutics. There are many commonalities between species, despite large differences in CRD1 sequence and overall stoichiometry. The receptor–ligand and C-terminal homodimerization interactions, as well as 1:1 receptor–ligand configurations are conserved. The C-terminal dimerization domain appears to drive signaling, as seen with a strong reduction of activity in the 3A9 IL-2 reporter assay with mutations that reduce the hydrophobic CRD3-CRD3 interactions observed. Furthermore, the C-terminal mediated receptor clustering may affect ligand binding, as the phenylalanine to alanine, arginine, or aspartic acid double mutants reduce overall levels of ligand staining seen in cell-imaging experiments.

This receptor homodimerization might lead to formation of higher-order structures on a cell membrane, engaging an intracellular network of TRAF molecules. We hypothesize the bent “C” shape of the receptor homodimers may point the transmembrane domain (TMD), and therefore also the intracellular domain, toward a neighboring TRAF molecule (Fig. S15). TNFRSF peptides bound within a trimeric TRAF are ~55 Å apart^35^, but hGITR C-termini in our structure have a distance of 108 Å, almost 40 Å more than other trimeric TNFRSF–TNFSF structures (Table S1). This would make intracellular peptides from an individual hGITR trimer spaced further apart than the optimal spacing for a single TRAF trimer to engage. If CRD3-CRD3 interactions are responsible for bringing together neighboring receptor trimers to form an intracellular TRAF network, it would be consistent with the diminished intracellular signaling we see in the IL-2 assay from mutations that reduce those interactions. These C-terminal interactions would also allow TMDs from neighboring GITR molecules to interact, the importance of which has been shown for other TNFR members, such as FAS, which forms trimers^32^, or DR5, which forms both dimers and trimers^36,37^. If GITR has dimeric TMD interactions, it might explain the residual clustering seen with the AA mutant.

For the mouse GITR–GITRL complex, it remains unknown how such linear arrangements of mGITR might engage with proposed models of hexagonal intracellular TRAF networks, perhaps requiring an adapter molecule as proposed for m4-1BB^38^. Previous experiments have reported increased activity for mGITRL C-terminal truncations in cell-based assays^25^. The similarities between species support the hypothesis that if a C-terminal mGITRL deletion leads to trimerization rather than strand-swapped dimers, it could engage mGITR as a trimer, leading to increased signaling. Taken together, this indicates the mGITR might not be signaling as strongly as hGITR or may require additional coadapters. As a result, preclinical murine in vivo studies with agonist antibodies that engage mGITR as a dimer might exhibit different behavior than agonist antibodies that engage trimeric human GITR. The structures and functional variants reported herein may lead to additional understanding of therapeutics and how extracellular GITR engages its ligand, driving intracellular signaling.

## Methods

### Expression and purification of GITR and GITRL proteins

DNA encoding residues 42–173, 20–153, 72–199, and 26–162 of murine GITRL (mGITRL), murine GITR (mGITR), human GITRL (hGITRL), and human GITR (hGITR), respectively, were cloned into an expression vector and used for transfection. To decrease heterogeneity, hGITR variants C57S, C58S, C66S, and C85S were tested, with C57S optimizing expression. mGITR and hGITR, residues 55 and 57, were mutated to serine (mGITR C55S and hGITR C57S) for further recombinant expression and purification. For cell assays, DNA encoding residues 74–199 of hGITRL with an N-terminal His tag were used in monomer form (His-hGITRL) or in the forced trimerization form with three copies of hGITRL each separated by GS linkers (His-hGITRL-sc).

All receptor and ligand proteins were generated by transient transfection into Expi293 cells (Thermo Fisher Scientific) with the addition of kifunensine (Enzo Life Sciences). Following 5–6 days of transient expression, the supernatant was harvested, filtered, and purified on an AKTA Avant (GE Healthcare). Supernatants were loaded onto a Ni Sepharose Excel column (GE Healthcare) to isolate recombinant proteins. His tags were removed by TEV cleavage overnight and the proteins were subsequently purified by SEC on either HiLoad 26/600 Superdex 75 or 200 pg (GE Healthcare) equilibrated in a buffer containing 25 mM Tris, pH 8.0, 100 mM NaCl. High mannose glycosylations were removed by endoglycosidase H according to the manufacturer’s instruction (Sigma Aldrich) prior to forming the receptor–ligand complexes. Mutant ligand proteins were expressed and purified in a similar manner without kifunensine and endo H treatment. To reconstitute the hGITR–hGITRL complex, the two proteins were mixed at receptor–ligand molar ratio of 5:3 with excess receptor, then subjected to SEC. The peak corresponding to the hGITR–hGITRL complex was collected for biophysical analysis and crystallization trials. Final buffer conditions were 10 mM Tris HCL, pH 8.0, 50 mM NaCl.

### SEC–MALS

The molecular weights of proteins were determined by using MALS. Protein samples (≥10 mg/mL, 50 μg) were injected onto an Acquity UPLC Protein BEH 125 or GE Superdex S200 5/150 GL column attached to a Waters Acquity UPLC H-Class system at a flow rate of 0.3 or 0.1 mL/min, respectively, in 1x PBS, 0.1% Na azide. The eluted peaks were analyzed using a Wyatt DAWN HELEOS-II MALS instrument and a Wyatt Optilab T-rEX differential refractometer. Data were re-evaluated in Astra v7.3.0 software using the Zimm model.

### Analytical ultracentrifugation

AUC was conducted using a Proteome Lab XL-I ultracentrifuge (Beckman Coulter) with an An-50 Ti rotor. Samples were loaded into two-channel sedimentation velocity cells in PBS. All runs were conducted at 20 °C with a rotor speed of 655,200 × *g*. Sedimentation was monitored at an absorbance of 280 nm. Data analysis was conducted with Sedfit v16.1c^39^ using a nonmodel-based continuous distribution corrected for time-invariant and radial-invariant noise. For theoretical coefficient comparisons, molecules were treated as compact spheres. Sedimentation coefficients were standardized to 20 °C in water.

### Structure determination

Crystals of hGITR–hGITRL were grown in sitting drops at 22 °C containing 1 μL protein and 1 μL well solution consisting of 30% PEG300, 0.2 M sodium sulfate decahydrate, and 0.1 M adenosine 5′-triphosphate disodium salt hydrate. Crystals of mGITR–mGITRL were grown by the same procedure but in a solution containing 17% PEG1500 and 0.1 M bicine, pH 8.0. Crystals were harvested at 1 week and cryoprotected with glycerol before flash freezing in liquid nitrogen. Diffraction data were collected at the Advanced Light Source beamline IMCA (17-ID) on a DECTRIS Pilatus 6M detector. All diffraction experiments were carried out 100 K. Data were collected at a wavelength of 1.0 Å. The crystal of hGITR–hGITRL complex belongs to the space group I23 with unit cell dimensions *a* = 168.42 Å, *b* = 168.42 Å, *c* = 168.42 Å, and diffracted to a resolution of 2.96 Å. The crystal of mGITR–mGITRL complex belongs to the space group P1211 with unit cell dimensions *a* = 70.54 Å, *b* = 64.82 Å, *c* = 81.23 Å, and diffracted to a resolution of 3.2 Å. Data of hGITR–hGITRL were processed with XDS. Data of mGITR–mGITRL were processed with Global Phasing autoPROC. The structure of hGITR–hGITRL was solved by molecular replacement using human GITR structure (PDB: 2Q1M) and an in-house generated human GITR model. The structure of mGITR–mGITRL was solved by molecular replacement using mouse GITRL structure (PDB: 2Q8O) and an in-house generated mouse GITR model. Alternate cycles of model building and refinement using the program Coot^40^ and model refinement using Phenix 1.15.2-3472 of coordinates and B factors with simulated annealing, NCS torsional restraints, and secondary structure restraints. Ramachandran statistics for hGITR–hGITRL complex are 94.43% favored and 0.94% outliers, for mGITR–mGITRL complex are 85.91% favored and 5.16% outliers. Figures and structural alignments and superpositions were generated using PyMOL. Final data collection and refinement statistics are listed in Table 1. Coordinates for the human and mouse structures are deposited in PDB (respectively, PDB IDs 7KHD and 7KHX).

### Cell line generation

3A9 cells (ATCC CRL-3293) were cultured in RPMI 1640 supplemented with 10% FBS, 1 mM Na pyruvate, and 10 mM Hepes. 3A9 cells were transfected with plasmids containing full-length GITR or full-length GITR mutants using a Lonza Nucleofector II. At 48 h post transfection, cells were placed under 800 μg/mL hygromycin selection. Following selection, the cells were screened by FACS and then sorted on a BioRad S3e Cell Sorter using Biolegend #371210 mouse anti-human CD357 (GITR) antibody (diluted 1:20). Receptor expression was validated with Biolegend #371210 or 6G10 (US20180164321A1) (diluted to 20 μg/mL) anti-GITR clones, and analyzed with a CytoFlex instrument (Beckman Coulter). Data were processed using FlowJo. Bulk sorted cells were then grown up in selection media for use in assays.

### GITR reporter assay

Secretion of mIL-2 from 3A9 cell lines was used to compare the activity of wild-type or mutant hGITRs upon His-hGITRL-sc stimulation. 2.5 × 10^4^ 3A9 cells expressing wild-type or mutant hGITRs were incubated in 96-well plates with 1 μg/mL of plate-bound anti-mCD3 (BD Biosciences #553058 or BioLegend #100340) and hGITR ligands at 37 °C. After 18–24 h, supernatants were harvested, and mIL-2 secretion was quantified by ELISA using commercial kits (BD Biosciences #555148 or R&D Systems #M2000). Absorbance was read on a Cytation 5 instrument (Agilent). Data were represented using GraphPad Prism v8.

### Confocal imaging

3A9 cells expressing human GITR or mutant hGITRs were cultured using RPMI with 10% FBS, 10 mM HEPES, pH 8.0, 0.1 M sodium pyruvate, and 800 μg/mL hygromycin B. To test for receptor clustering, 2.0 × 10^4^ 3A9 cells were aliquoted into wells in the presence of biotinylated Avi-His-hGITRL-sc for 15 min on ice (nonbiotinylated Avi-His-hGITRL-sc was used to check background signal). Cells were then fixed in 4% paraformaldehyde in 1x PBS for 10 min at room temperature. Cells were then washed in 1x PBS and stained with streptavidin, Alexa Fluor 488 conjugate (Thermo Fisher) and Hoechst 33342 (Thermo Fisher) for 30 min on ice. Cells were washed twice with 1x PBS and transferred to a 96-well PDL-coated CellCarrier Ultra plate (Perkin Elmer), fixed with 3.8% formaldehyde, and immediately centrifuged at 524 × *g* for 1 min and left for 15 min prior to imaging. Cells were imaged using the Operetta CLS High-Content Analysis System (Perkin Elmer) at a 63× water objective and 12 Z-stack Max projection. Imaging quantification was performed with Harmony (Perkin Elmer), using method C cytoplasm detection and method B spot detection.

## Supplementary information

Supplementary Information

## Data Availability

The crystal structures reported in this manuscript are available from the Protein Data Bank under accession codes 7KHD (human GITR–GITRL complex) and 7KHX (mouse GITR–GITRL complex). Source data are provided with this paper. Other data reported in this manuscript are available from the corresponding author upon reasonable request.

## Source data

Source Data
