# Supplementary material for: Structures of mouse and human GITR–GITRL complexes reveal unique TNF superfamily interactions
Source: Nat Commun. 2021 Mar 2;12:1378. doi: 10.1038/s41467-021-21563-z (PMC7925557; doi:10.1038/s41467-021-21563-z)
Supplement: Supplementary file 1 — Supplementary Information [file 41467_2021_21563_MOESM1_ESM.pdf]

Supplementary Information

Structures of Mouse and Human GITR-GITRL Complexes Reveal Unique TNF Superfamily Interactions

Feng Wang*, Bryant Chau*, Sean M. West*, Christopher R. Kimberlin, Fei Cao, Flavio Schwarz, Barbara Aguilar, Minhua Han, Winse Morishige, Christine Bee, Gavin Dollinger, Arvind Rajpal, and Pavel Strop#

*Authors contributed equally, #corresponding author

Supplementary Figures

Supplementary Figure 1. Multiple sequence alignment of mGITR and hGITR with non-conserved cysteine locations highlighted on structure.
Supplementary Figure 2. Stereo images of the structures and 2Fo-Fc electron density.
Supplementary Figure 3. Multiple sequence alignment across species of the interface of GITRL and GITR.
Supplementary Figure 4. Comparison of the unbound and bound GITRL structures.
Supplementary Figure 5. Crystallographic packing of the asymmetric unit of hGITR-hGITRL.
Supplementary Figure 6. SEC-MALS and analytical SEC data for human and mouse GITR and GITRL monomers and complexes.
Supplementary Figure 7. Affinity and avidity driven binding of GITR to GITRL.
Supplementary Figure 8. Sedimentation coefficients for human GITR, human GITRL and human GITR-GITRL complex as measured by analytical ultracentrifugation.
Supplementary Figure 9. FACS analysis of hGITR cell lines.
Supplementary Figure 10. Confocal cell imaging of WT and F106S cells with anti-GITR Fab.
Supplementary Figure 11. Confocal cell imaging of WT, AA, RR, and DD cells with anti-GITR Fab and hGITRL-sc.
Supplementary Figure 12. Comparison of hGITR CRD1, CRD2, and CRD3 domains to other TNFRSF family members.
Supplementary Figure 13. Comparison of mGITRL and m4-1BBL dimer rotation upon receptor binding.
Supplementary Figure 14. Comparison of GITR and TNRF1 dimer assemblies.
Supplementary Figure 15. Proposed model of GITR C-terminal interactions.

Supplementary Tables

Supplementary Table 1. Distance between C-terminal ends of TNFRs bound to ligand trimers.

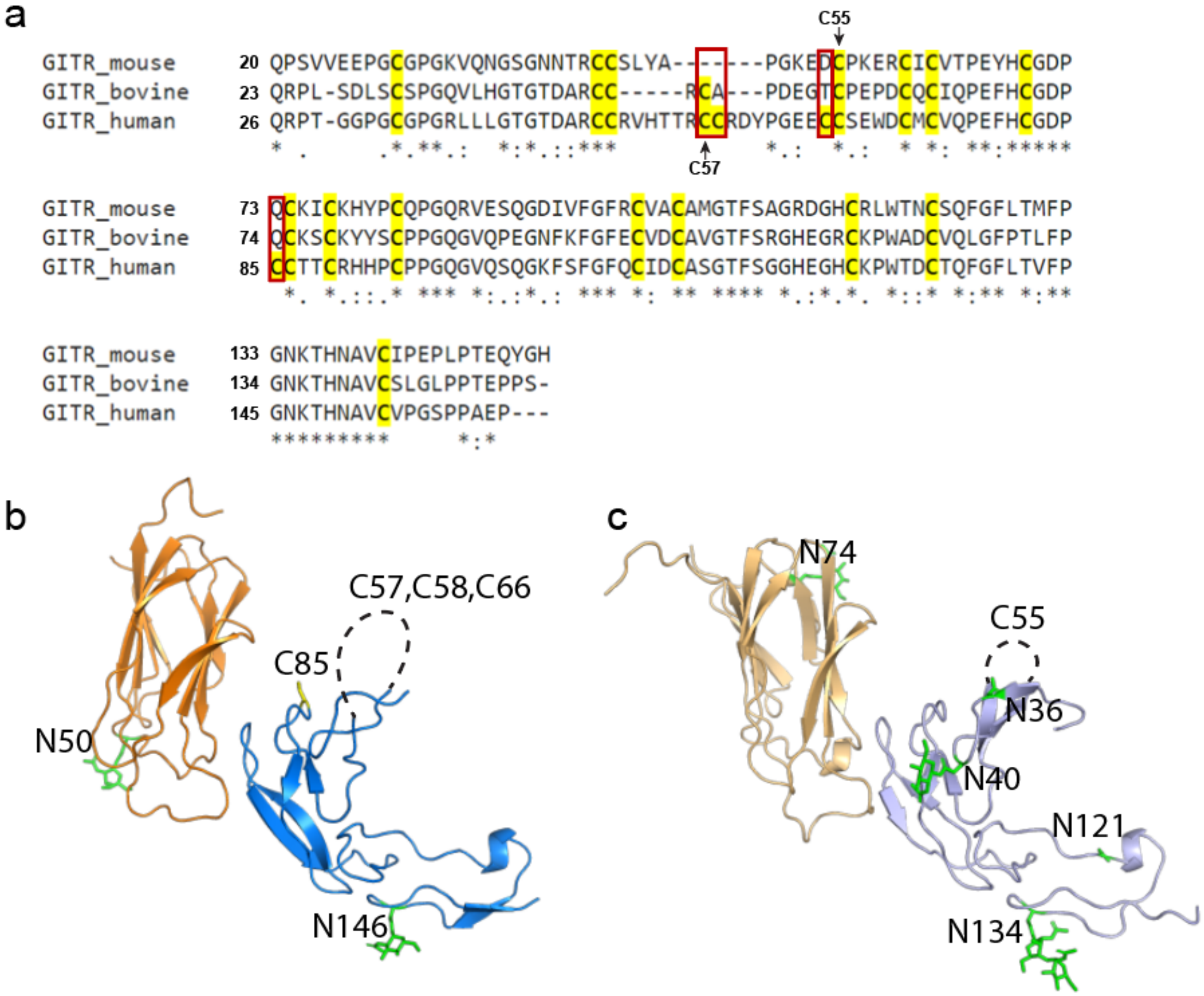

Supplementary Figure 1. Multiple sequence alignment (a) of the extracellular domain of human, mouse, and bovine GITR, cysteines highlighted in yellow, non-conserved cysteines boxed. hGITR C57, and mGITR C55 indicated with arrows. Multiple sequence alignment showed that 15 cysteines are highly conserved through human, mouse, and bovine, leaving the 4 non-conserved cysteines as candidates to be individually mutated to serine (C57S, C58S, C66S or C85S) for optimizing expression. All four hGITR mutants were tested for expression. The positions of the cysteines that were mutated for human (b) and mouse (c) are shown on the structure (receptor shown in blue, ligand shown in orange), along with the position of the putative glycosylation sites (shown in green, glycosylation on mGITR was only resolved for N40 and N134 in our structure).

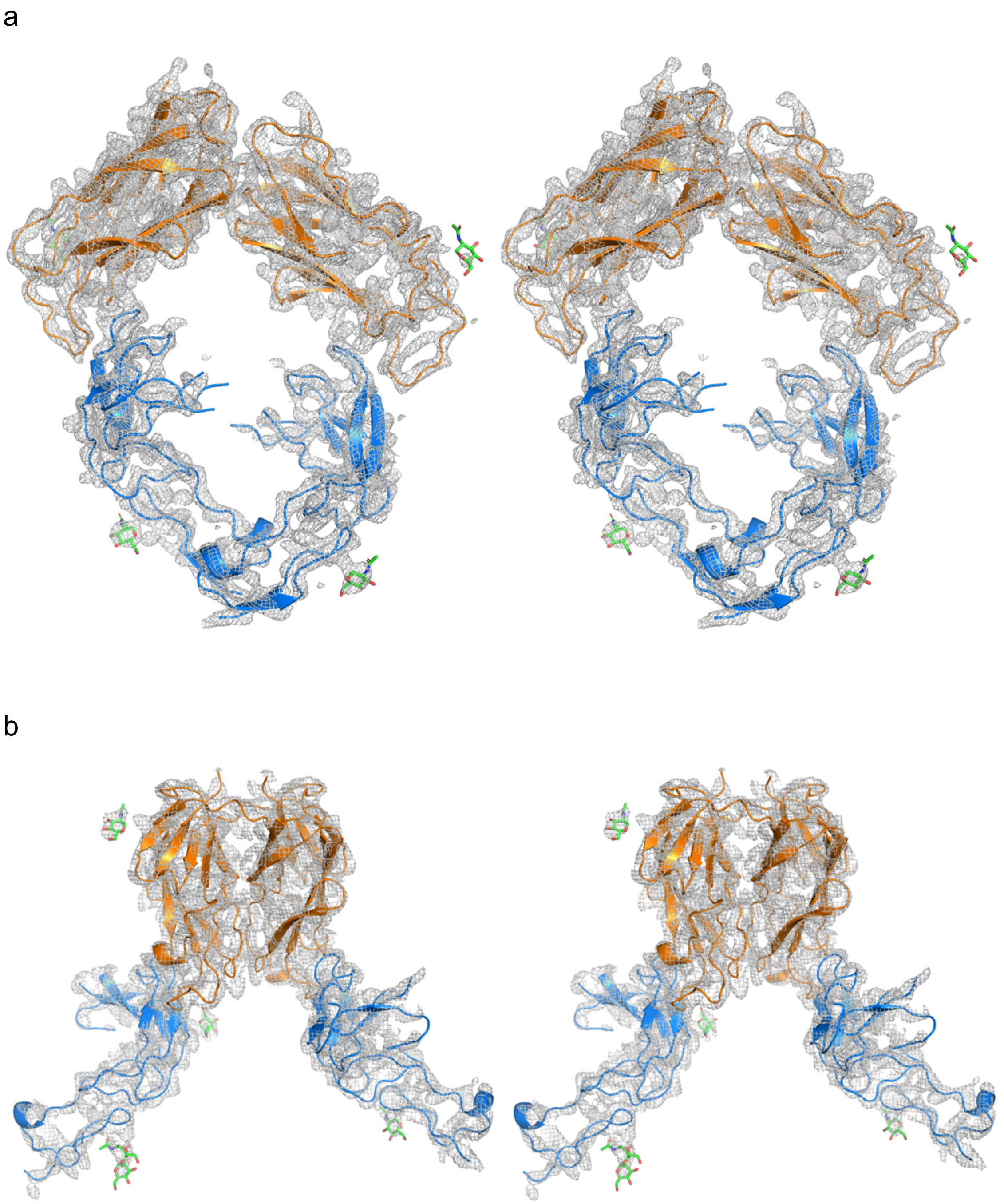

Supplementary Figure 2. Stereo images of the structures and 2Fo-Fc electron density maps (contoured at 1.5 sigma) for human (a) and mouse (b) receptor-ligand complexes (receptor shown in blue, ligand in orange, glycans in green).

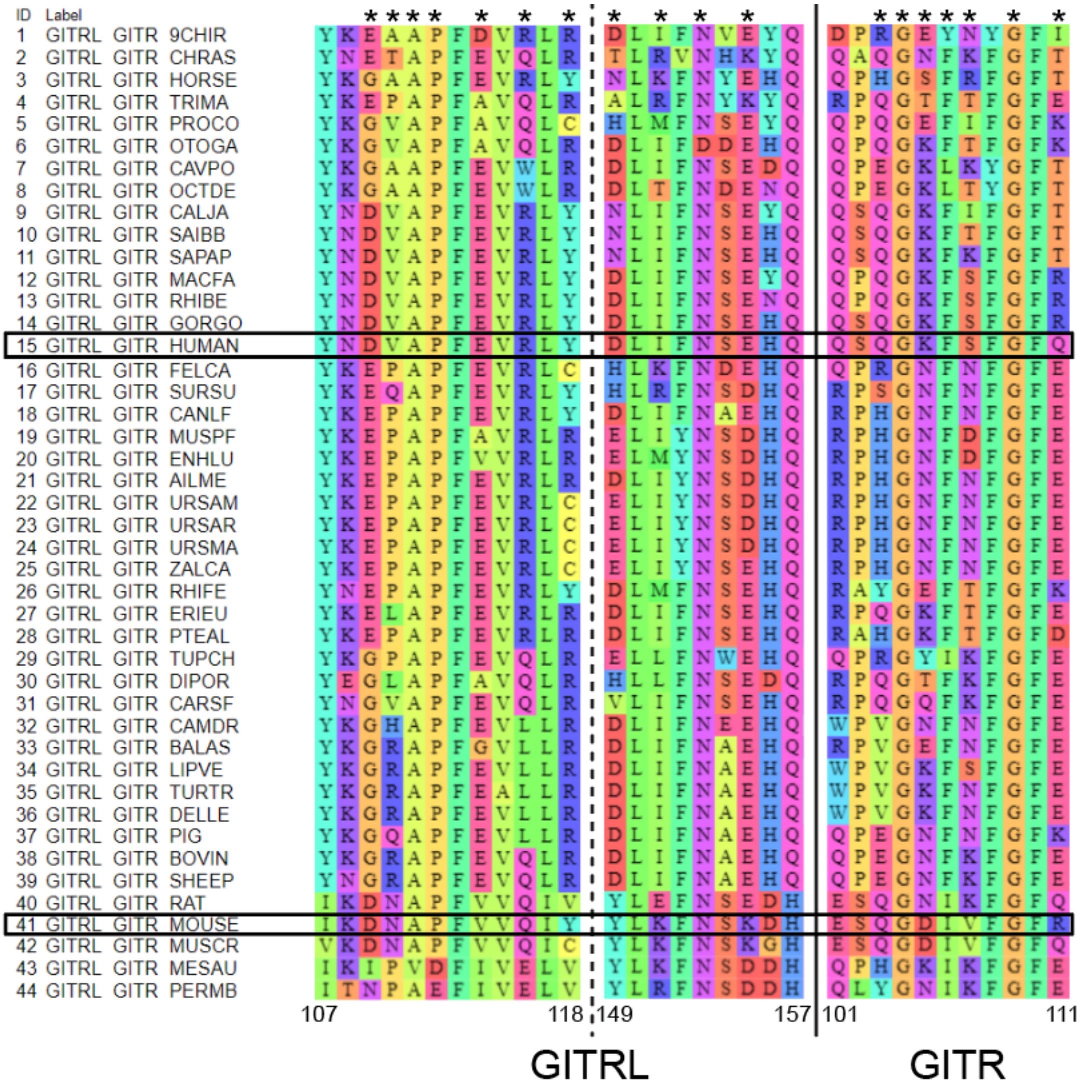

Supplementary Figure 3. Multiple sequence alignment across species of the interface of GITRL and GITR, numbering is provided for the human ligand and receptor. Residues which are near the interface of the two crystal structures, which could potentially be involved in interactions are marked with an asterisk. Human and mouse sequences are boxed.

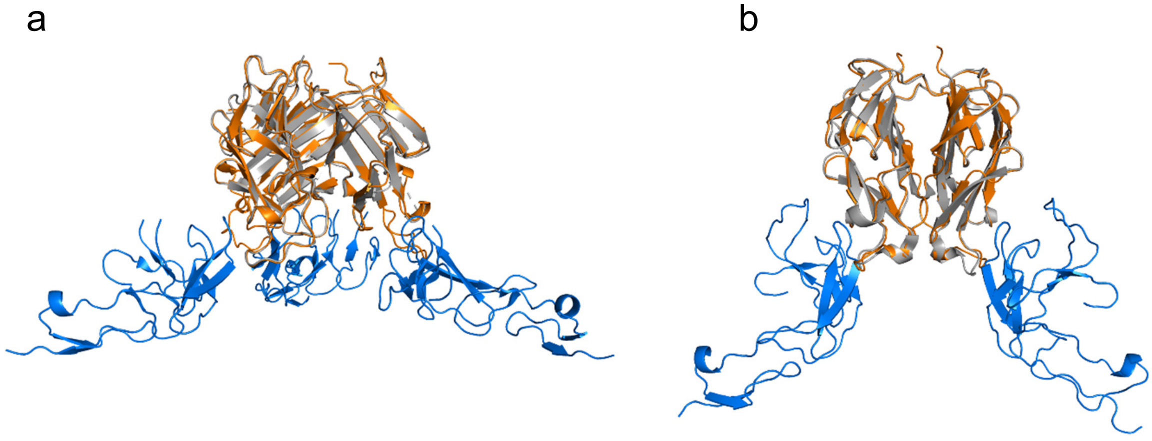

Supplementary Figure 4. Comparison of the unbound human (a) and mouse (b) GITRL complexes (PDB 3B93 and 2QDN respectively) in grey, overlaid with the bound ligand-receptor complexes (blue and orange) show no conformational change in GITRL upon binding.

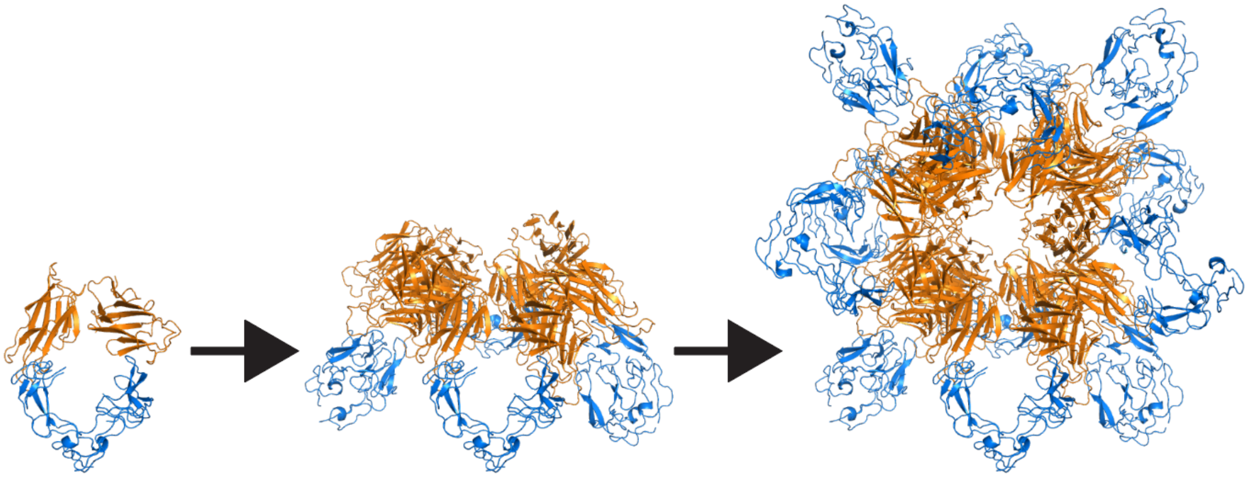

Supplementary Figure 5. Crystallographic packing of the asymmetric unit in the hGITR-hGITRL (GITR in blue, GITLR in orange) crystal structure has higher order structures that are unlikely to be found on cell surfaces due to their pronounced curvature.

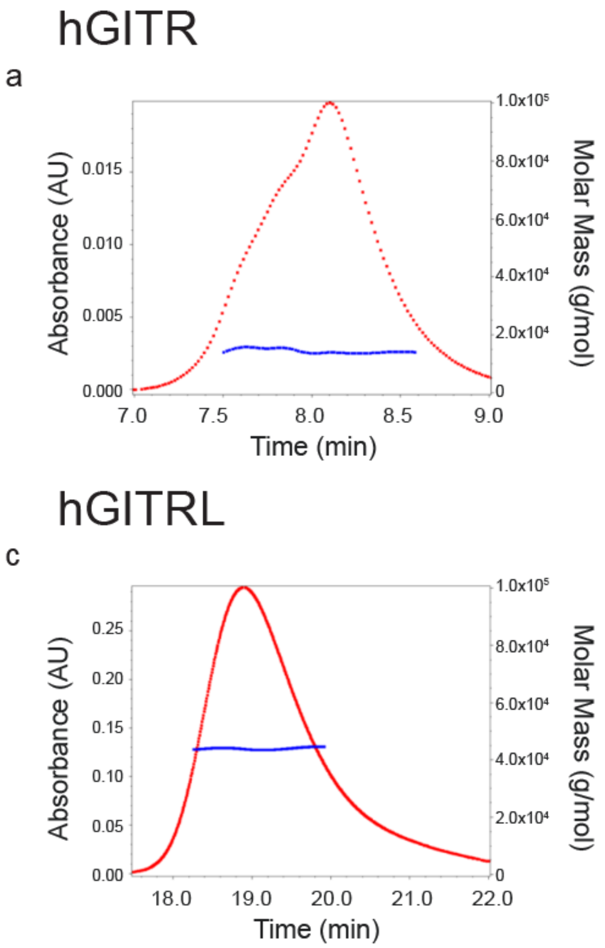
hGITR
a
mGITR
b
hGITRL
c
mGITRL
d
Absorbance (AU)
Molar Mass (g/mol)
Time (min)
0.000
0.005
0.010
0.015
7.0
7.5
8.0
8.5
9.0
0.00
0.05
0.10
0.15
0.20
0.25
18.0
19.0
20.0
21.0
22.0
23.0
0
2.0x10⁴
4.0x10⁴
6.0x10⁴
8.0x10⁴
1.0x10⁵

e

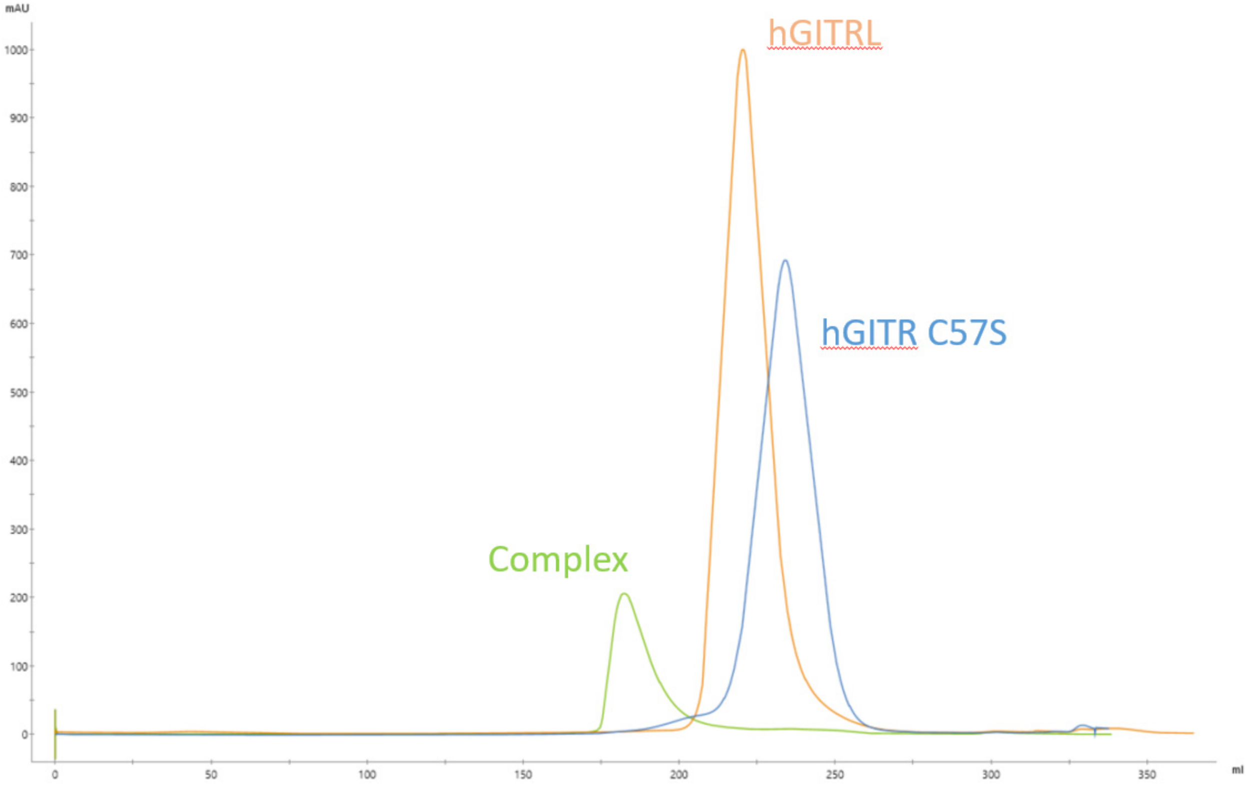
mAU
hGITRL
hGITR C57S
Complex
ml
0
50
100
150
200
250
300
350
100
200
300
400
500
600
700
800
900
1000

f

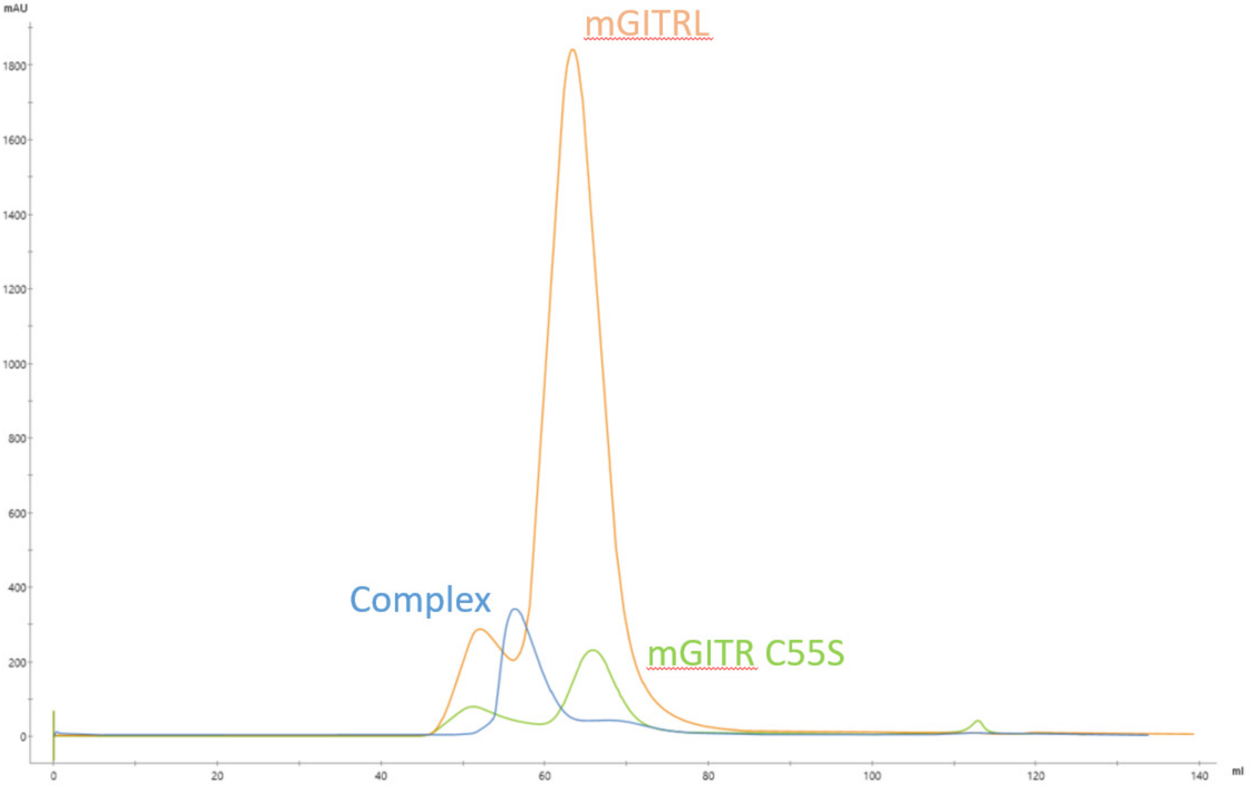
mAU
mGITRL
Complex
mGITR C55S
ml
0
20
40
60
80
100
120
140
200
400
600
800
1000
1200
1400
1600
1800

Supplementary Figure 6. SEC-MALS analysis of human and mouse GITR and GITRL. Analysis of hGITR (a) and mGITR (b) by SEC-MALS gives an experimental molar mass of 14 and 19 kDa respectively, closely matching the calculated masses of 14.6 kDa and 16.9 kDa for the two receptors. Analysis of hGITRL (c) and mGITRL (d) by SEC-MALS gives an experimental molar mass of 45 kDa and 38 kDa respectively. The experimental mass of hGITRL matches the predicted mass of a 45 kDa trimer and mGITRL dimer with predicted 35 kDa molar mass. For SEC-MALS experiments, hGITR retention time was determined on Waters BEH125 column, while retention times of other proteins (b-d) were determined on GE Superdex S200. Preparative size exclusion profiles of hGITR-hGITRL (e) and mGITR-mGITRL (f) complex overlaid with the individual components. Retention time of hGITRL-hGITRL complex and individual components (e) were determined on GE Superdex S200. Retention time of mGITR-mGITRL complex and individual components (f) were determined on GE Superdex S75.

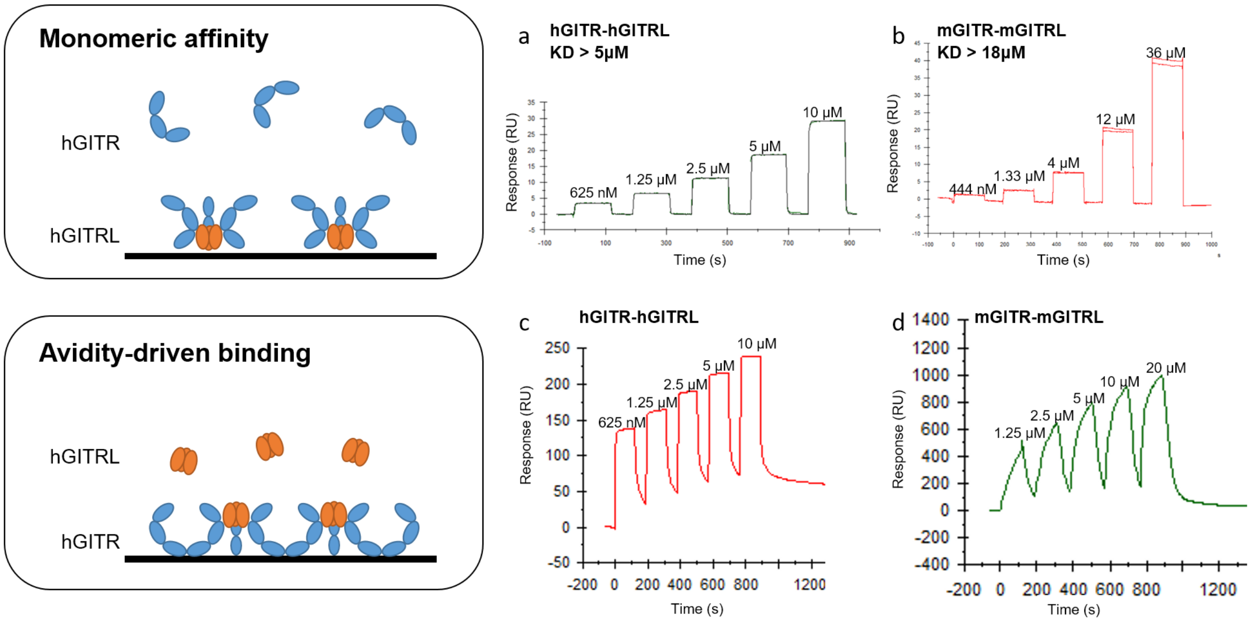

Supplementary Figure 7. With GITRL immobilized (a,b) to give monomeric affinity, GITR is flowed over chip at 5 different concentrations to measure 1:1 affinity. Both human (a) and mouse (b) receptor-ligand complexes show poor (> 5 μM and >18 μM) 1:1 affinity. With GITR immobilized (c,d), binding to the receptor is driven by avidity of the trimeric human ligand (c) or the dimeric mouse ligand (d).

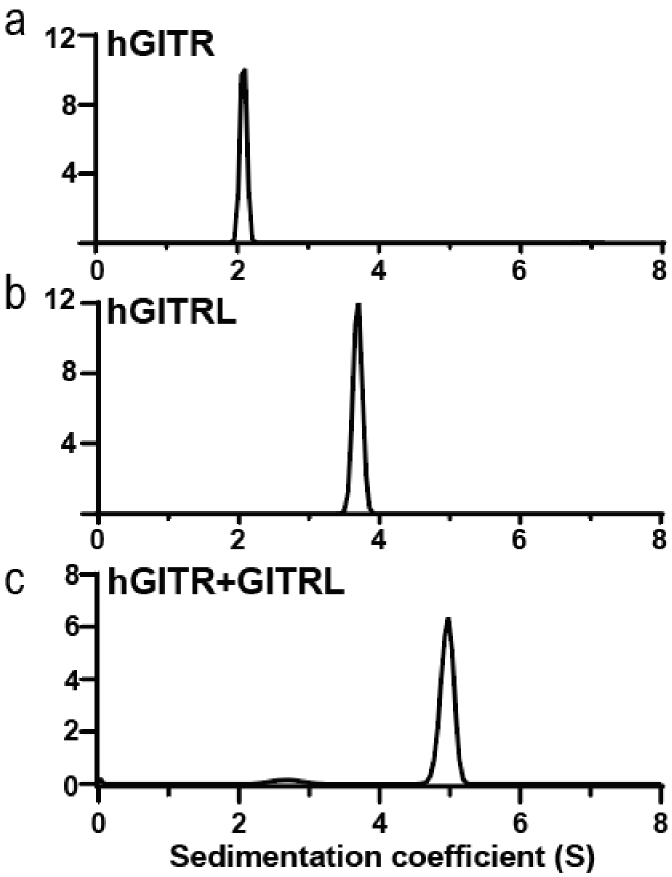

Supplementary Figure 8. Sedimentation coefficients for human GITR, human GITRL and human GITR-GITRL complex as measured by analytical ultracentrifugation. We analyzed soluble hGITR (a), hGITRL (b), and hGITR-GITRL complex (c) by sedimentation velocity on an analytical ultracentrifuge (AUC). hGITR monomer sediments as a single peak at 2.14S (predicted sedimentation coefficient from bead model is 2.3S). hGITRL trimer sediments as a single peak at 3.7S (predicted coefficient 3.3S). The hGITR-hGITRL complex (c) contains a large peak at 4.98S, matching the predicted coefficient for the 3:3 receptor-ligand complex, and a small peak of excess hGITR at 2.7S.Variability from predicted coefficients may be due to experimental molecules (f/f_0 = 1.05-1.35) deviating from a compact sphere (f/f_0 = 1).

a

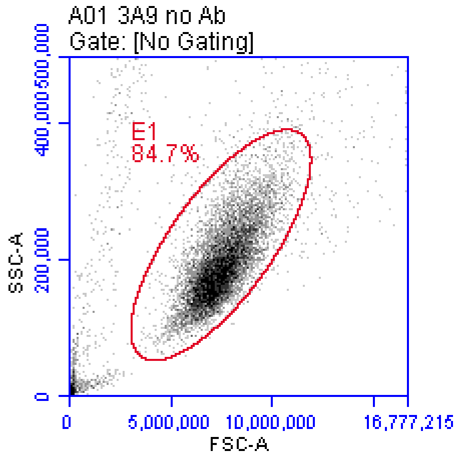

b

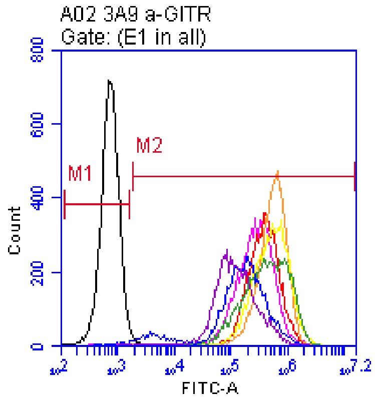

c

Plot 5: Multiple Samples	Count	Volume (µL)	% of This Plot	% of All	Mean FITC-A	CV FITC-A
**Gated on (E1 in all)**						
A02 3A9 a-GITR : This Plot	8,636	10	100.00%	86.36%	761.46	38.27%
A02 3A9 a-GITR : M1 (107.0 / 1,633.0)	8,536	10	98.84%	85.36%	747.65	34.04%
A02 3A9 a-GITR : M2 (1,754.0 / 16,777,215.0)	64	10	0.74%	0.64%	2,156.52	28.41%
A03 3A9 GITR WT a-GITR : This Plot	8,087	6	100.00%	80.87%	353,807.15	73.69%
A03 3A9 GITR WT a-GITR : M1 (107.0 / 1,633.0)	4	6	0.05%	0.04%	845.25	42.10%
A03 3A9 GITR WT a-GITR : M2 (1,754.0 / 16,777,215.0)	8,082	6	99.94%	80.82%	354,025.40	73.63%
A04 3A9 GITR 104V a-GITR : This Plot	7,152	7	100.00%	71.52%	459,685.40	62.71%
A04 3A9 GITR 104V a-GITR : M1 (107.0 / 1,633.0)	0	7	0.00%	0.00%	0	0.00%
A04 3A9 GITR 104V a-GITR : M2 (1,754.0 / 16,777,215.0)	7,152	7	100.00%	71.52%	459,685.40	62.71%
A05 3A9 GITR 106S a-GITR : This Plot	8,163	7	100.00%	81.63%	643,668.02	50.85%
A05 3A9 GITR 106S a-GITR : M1 (107.0 / 1,633.0)	0	7	0.00%	0.00%	0	0.00%
A05 3A9 GITR 106S a-GITR : M2 (1,754.0 / 16,777,215.0)	8,163	7	100.00%	81.63%	643,668.02	50.85%
A06 3A9 GITR 109D a-GITR : This Plot	7,907	7	100.00%	79.07%	654,816.53	67.87%
A06 3A9 GITR 109D a-GITR : M1 (107.0 / 1,633.0)	1	7	0.01%	0.01%	767	0.00%
A06 3A9 GITR 109D a-GITR : M2 (1,754.0 / 16,777,215.0)	7,906	7	99.99%	79.06%	654,899.26	67.86%
B01 3A9 GITR AA a-GITR : This Plot	7,711	6	100.00%	77.11%	606,278.26	79.55%
B01 3A9 GITR AA a-GITR : M1 (107.0 / 1,633.0)	0	6	0.00%	0.00%	0	0.00%
B01 3A9 GITR AA a-GITR : M2 (1,754.0 / 16,777,215.0)	7,711	6	100.00%	77.11%	606,278.26	79.55%
B02 3A9 GITR DD a-GITR : This Plot	6,561	7	100.00%	65.61%	228,843.90	99.06%
B02 3A9 GITR DD a-GITR : M1 (107.0 / 1,633.0)	59	7	0.90%	0.59%	1,176.12	25.67%
B02 3A9 GITR DD a-GITR : M2 (1,754.0 / 16,777,215.0)	6,492	7	98.95%	64.92%	231,262.87	98.02%
B03 3A9 GITR RR a-GITR : This Plot	6,214	5	100.00%	62.14%	175,146.95	95.76%
B03 3A9 GITR RR a-GITR : M1 (107.0 / 1,633.0)	4	5	0.06%	0.04%	1,400.75	12.74%
B03 3A9 GITR RR a-GITR : M2 (1,754.0 / 16,777,215.0)	6,210	5	99.94%	62.10%	175,258.86	95.69%

Supplementary Figure 9. FACS analysis of hGITR cell lines generated shows high receptor expression across WT and mutant cell lines. (a) Initial gating performed using SSC-A vs. FSC-A for singlets. (b) Cell lines sorted by anti-GITR (Biolegend #371210) binding. Receptor mutants at the ligand interface F106S and G109D, as well as receptor-receptor mutants hGITR-AA, and hGITR-RR and hGITR-DD have comparable expression as measured by FACS. (c) Number of cells in Parental = 8636, WT=8087, F106S=8163, G109D=7907, AA=7711, RR=6214, DD=6561.

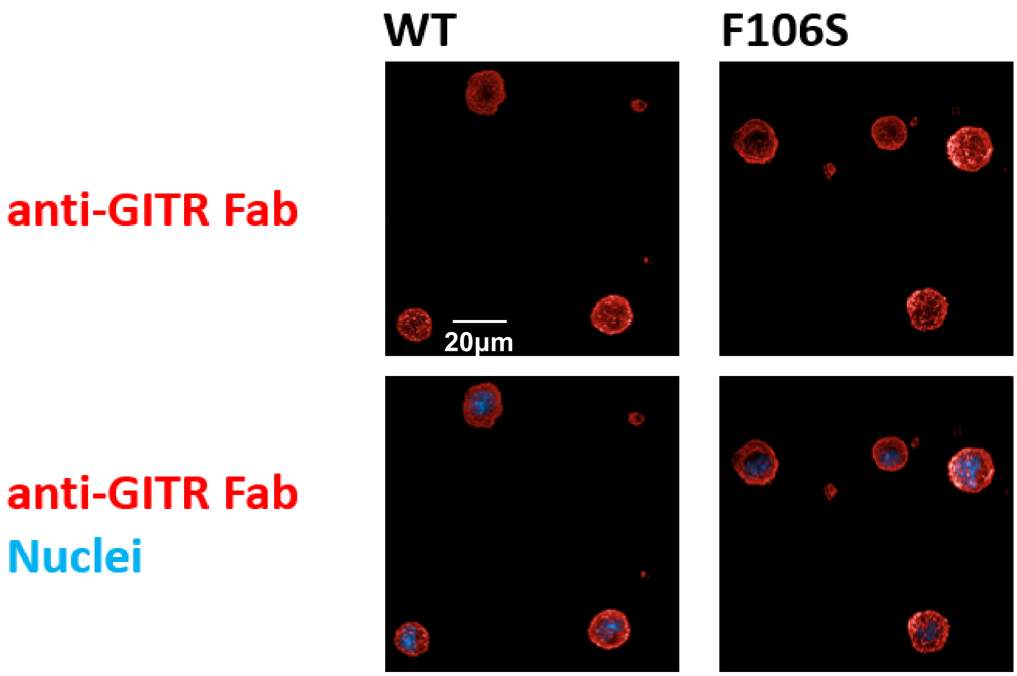

Supplementary Figure 10. Confocal cell imaging using anti-GITR Alexa Fluor 647 Fab staining (4 µg/mL) showed comparable expression level of GITR in both wild type and F106S mutant cell lines. This imaging experiment was performed once, cells were plated in duplicate.

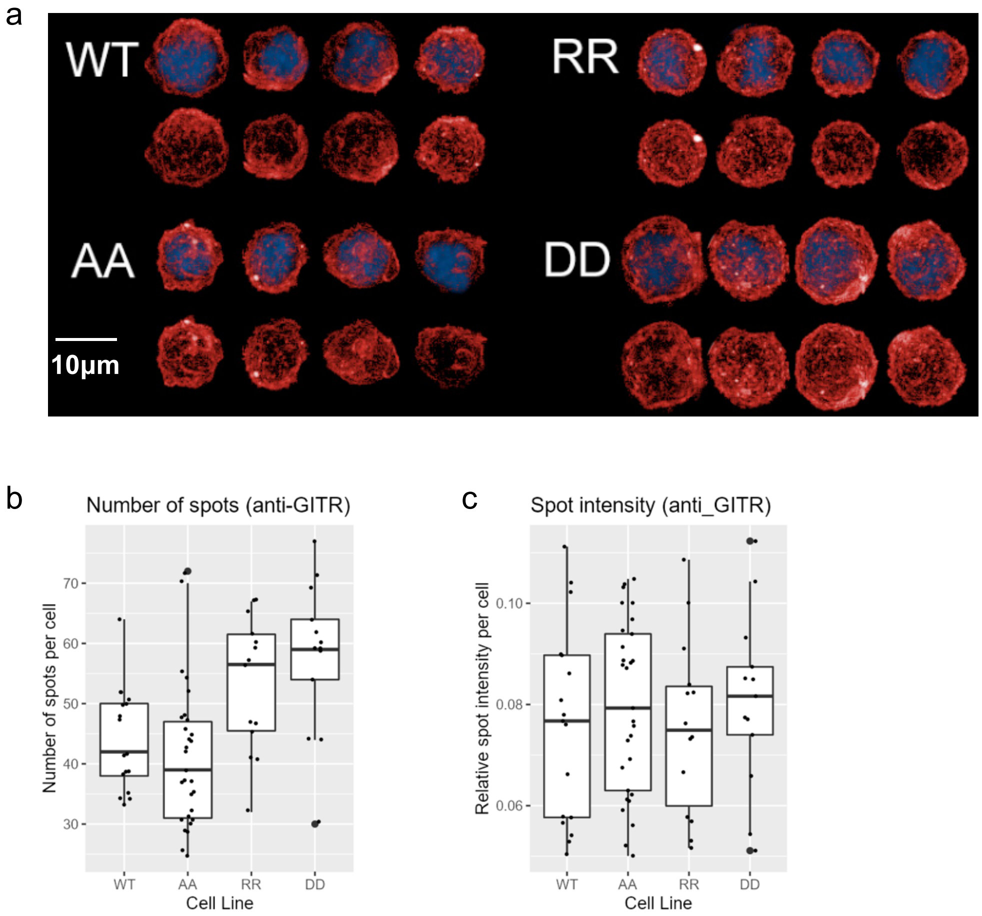

a
WT
RR
AA
DD
10μm
b
Number of spots (anti-GITR)
Number of spots per cell
70
60
50
40
30
WT
AA
RR
DD
Cell Line
c
Spot intensity (anti_GITR)
Relative spot intensity per cell
0.10
0.08
0.06
WT
AA
RR
DD
Cell Line

d

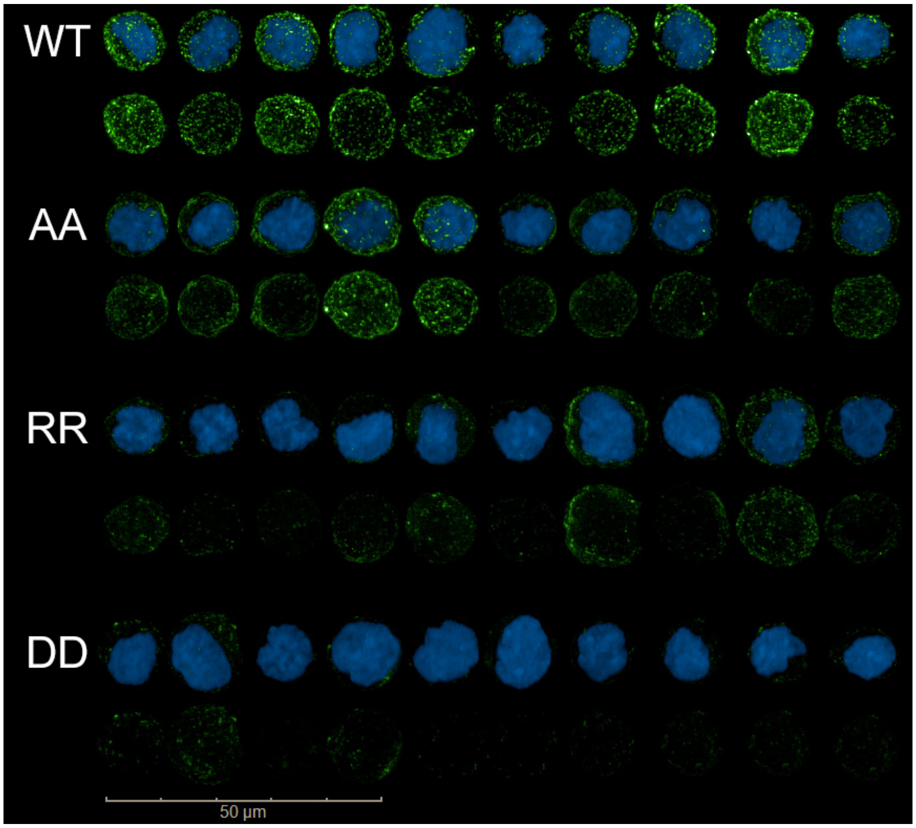

e

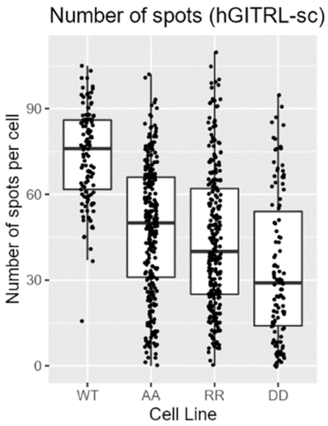

f

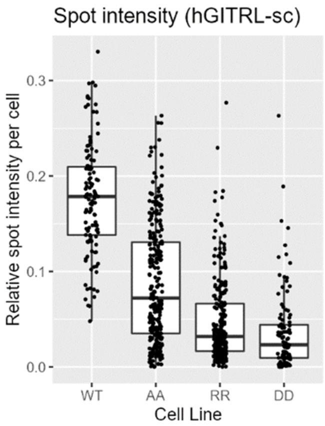

Supplementary Figure 11. (a) Confocal cell imaging of CRD3 AA, RR, and DD mutants show similar receptor expression compared to WT using anti-GITR Alexa Fluor 647 Fab staining (5 µg/mL), this imaging experiment was performed once, cells plated in duplicate. Quantification receptor density by anti-GITR staining shows all mutant lines have at least as many spots per cell (b), as well as equal spot intensity (c) to the WT cell line (number cells WT=17, AA=29, RR=14, DD=13). (d) Imaging shows CRD3 mutants (AA, RR, DD) reduce trimeric hGITRL-sc (0.125 µg/mL) binding on cells, this imaging experiment was performed once, cells plated in duplicate. Quantification of hGITRL-sc binding shows reduced number of spots (e) and reduced spot intensity (f) (number cells WT=120, AA=268, RR=253, DD=110). Median (50%) and quartiles (25%, 75%) shown in boxplots with individual data points, upper and lower whiskers represent furthest point within 1.5 * interquartile (25% - 75%) range.

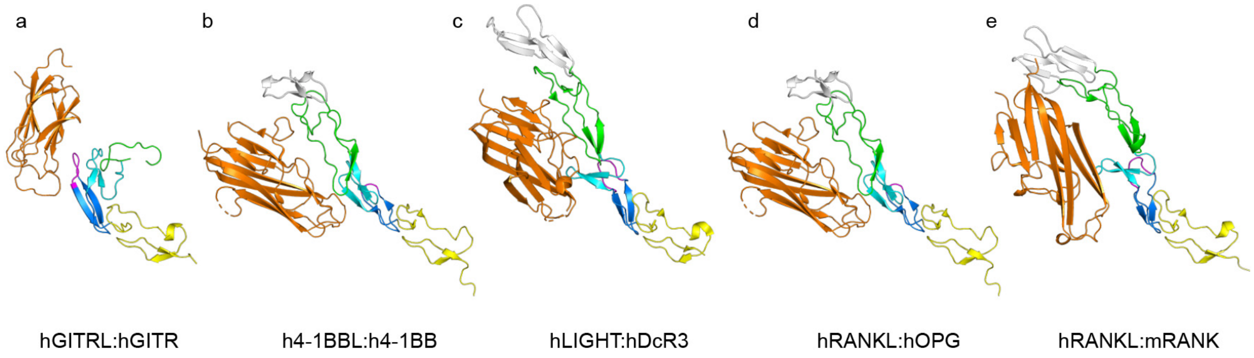

Supplementary Figure 12. Comparison of hGITR CRD1 (green), CRD2 (cyan and blue), and CRD3 (yellow) domains aligned in the CRD3 region to other TNFRSF members with similar structure as determined by DALI[1] (h4-1BB [6A3V], hDcR3 [4J6G], hOPG [3URF], mRANK [5BNQ]). The hGITR CRD2 B1 (blue) module that interacts with GITRL is similar to some TNFRSF members, but is extended and interacts with its ligand (magenta loop). This module (blue with magenta loop) does not make ligand interactions in other TNFRSF members shown. The CRD2 A2 module of hGITR that precedes B1 (cyan) does not structurally align with other TNFRSF members, giving its CRD2 domain a unique topology; this A2 module (cyan) interacts with the TNF ligands in other members shown.

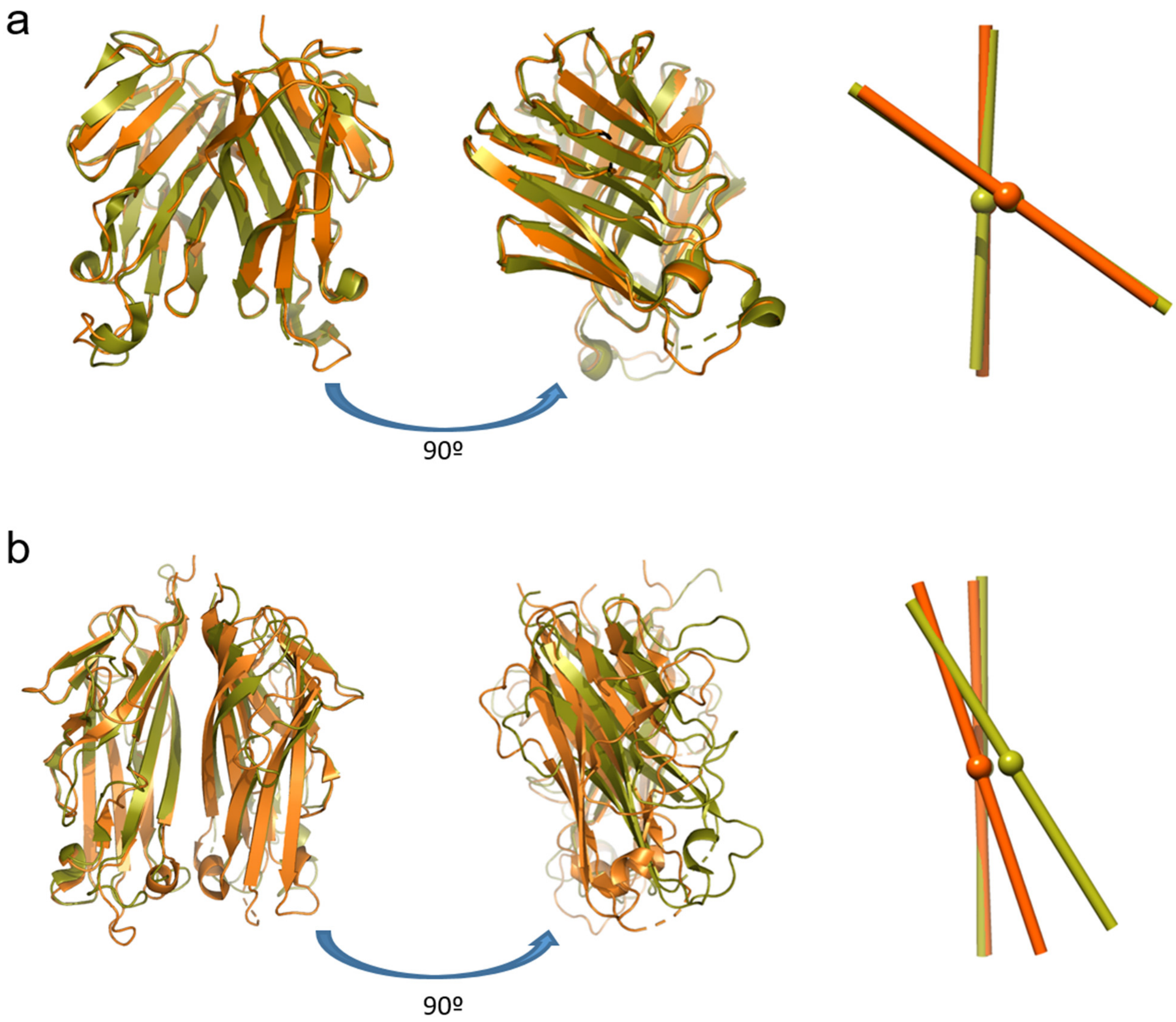

Supplementary Figure 13. Comparison of mouse GITRL (a) and mouse 4-1BBL (b) dimer rotation upon receptor binding. The receptor bound dimer are shown in orange cartoon and apo dimers are shown in olive cartoons on the left. Calculated ligand protomer center of mass (spheres) and long axis (rods) are the right showing the change in relative rotation between ligands with receptor binding.

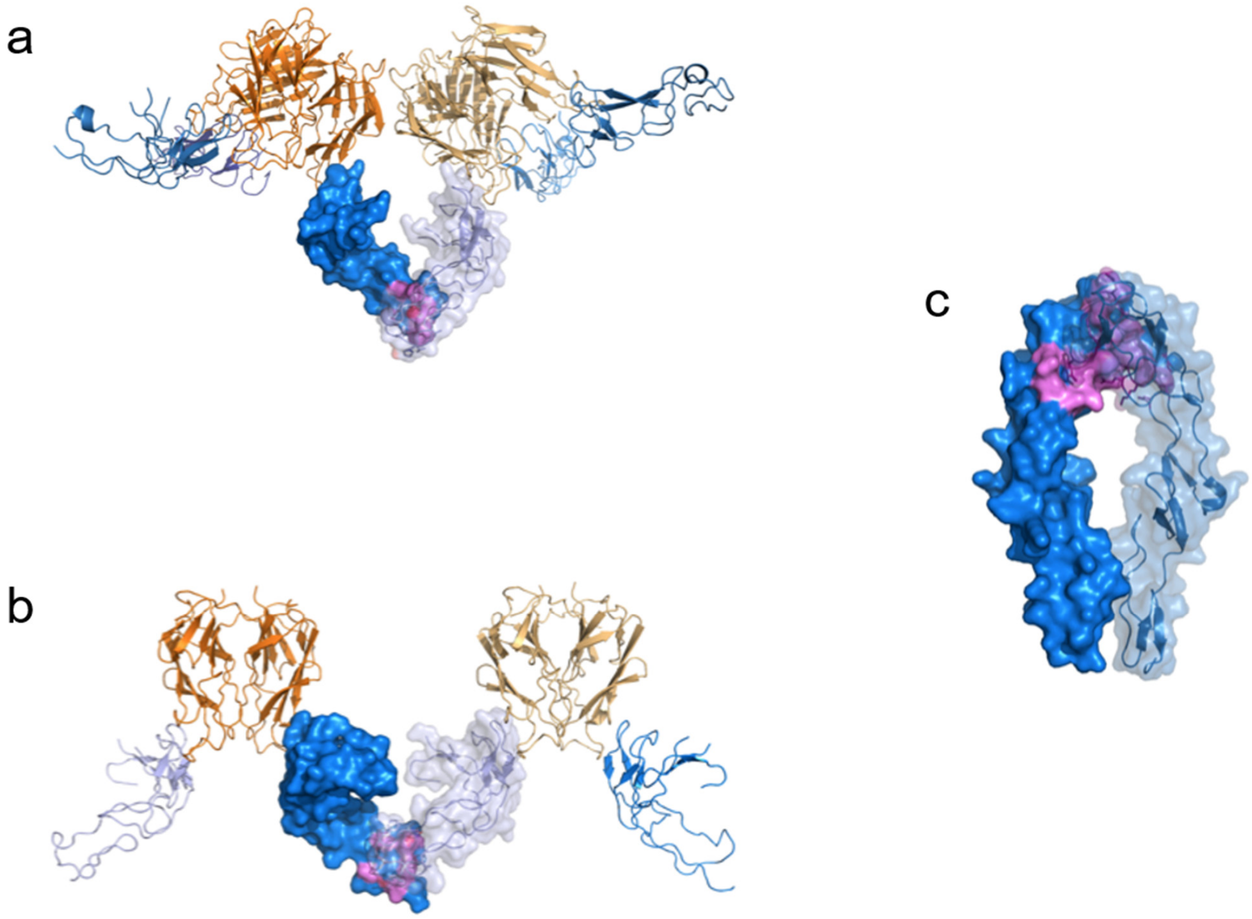

Supplementary Figure 14. Comparison of TNFR dimer assemblies: hGITR (a) and mGITR (b) dimers highlighted with critical interface residues shown in violet. (c) TNRF1 (1NCR) parallel dimer with PLAD interface highlighted in violet.

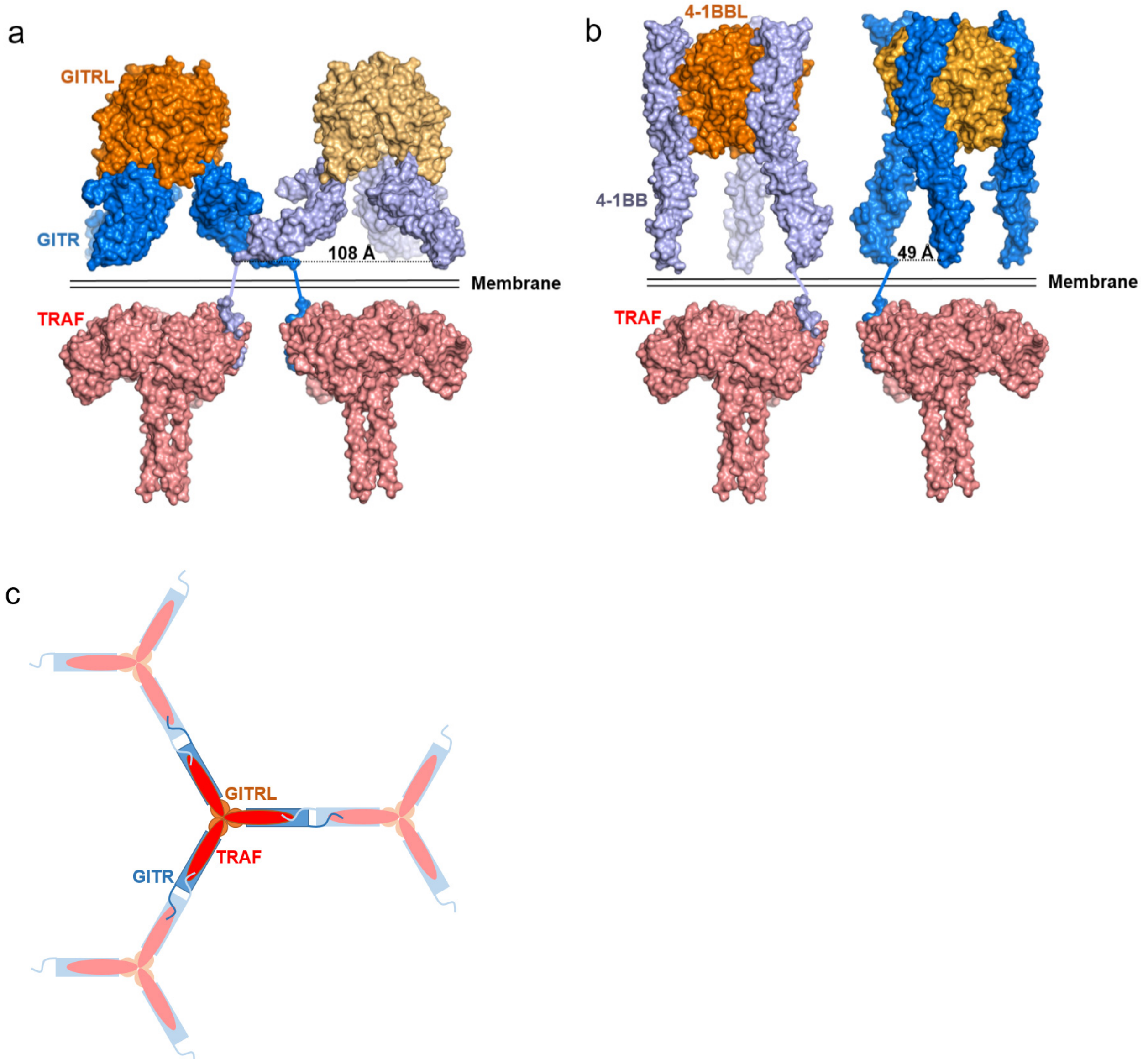

Supplementary Figure 15. Proposed model of C-terminal interactions of TNFSFR using structure of TRAF bound to C-terminus of TNFR (PDB ID: 1CA9). (a) The "hexameric" model of two hGITR-hGITRL trimers position the membrane proximal C-termini of GITR ECD towards an intracellular TRAF trimer adjacent to the GITR trimer it binds. This arrangement is possible due to the unique splayed GITR structure with C-terminal homodimeric interactions, the C-termini of GITR within a trimeric complex are approximately 108 Å apart. (b) A model of 4-1BB:4-1BBL (6MGP) interacting with TRAF shows that with 49 Å between C-termini of receptors within a trimer, a receptor is able to directly interact with a TRAF trimer without interacting with adjacent receptors or TRAFs. (c) Model of a higher order GITR-TRAF network shows TRAF trimers bridging flanking GITR trimers.

Receptor	C-termini spacing (Å)	ECD to TM linker length (AA)
hGITR	108 Å	7
OX40 (2HEV)	71 Å	46
TNFR2 (3ALQ)	62 Å	57
4-1BB (6MGP)	49 Å	24
CD40 (3QD6)	47 Å	48
BCMA (1XU2)	45 Å	11
TACI (1XU1)	41 Å	56
DR5 (1DU3)	41 Å	27
TNFR1 (1TNR)	32 Å	29

Supplementary Table 1. Distance between C-terminal ends of TNFRs bound to ligand trimers, and the number of amino acids between the structured ECD and the transmembrane (TM) domain. The hGITR structure reported here has a distance of almost 40 Å more between membrane-proximal compared to other trimeric TNFR-ligand structures, with the shortest linker between the receptor and the membrane (only 7 amino acids).

1. Holm L. DALI and the persistence of protein shape. *Protein Sci* **29**, 128-140 (2020).
